# Supplementary material for: Conformational plasticity of a BiP–GRP94 chaperone complex
Source: Nat Struct Mol Biol. 2025 Jul 14;32(10):1947–58. doi: 10.1038/s41594-025-01619-0 (PMC12527940; doi:10.1038/s41594-025-01619-0)

nature structural & molecular biology

Supplementary information https://doi.org/10.1038/s41594-025-01619-0

Conformational plasticity of a BiP–GRP94 chaperone complex

In the format provided by the authors and unedited

Supplementary Table 5. Primers used for molecular cloning

Point mutation	Forward Primer	Reverse Primer	Plasmid
**Primers used for site-directed mutagenesis of His6-BiP in pNIC28-Bsa4**			
BiP E243K	CTTCTCACCATTCGCAATGGTGTC	GACACCATTGCGAATGGTGAGAAG	pNIC28-Bsa4
BiP I437V	GACCAAACTGGTTCCAAGGAACAC	GTGTTCCTTGGAACCAGTTTGGTC	pNIC28-Bsa4
BiP V461F	GATAATCAACCAACTTTTACAATCAAGGTCTATGAA	TTCATAGACCTTGATTGTAAAAGTTGGTTGATTATC	pNIC28-Bsa4
**Primers used for site-directed mutagenesis of Strep-GRP94 FL in pET21d**			
GRP94 E103A	GATTTTCCTGAGAGCACTGATTTCAAATGC	GCATTTGAAATCAGTGCTCTCAGGAAAATC	pET21d
GRP94 D149N	GCATGTCACAAACACGGGTGTAG	CTACACCCGTGTTTGTGACATGC	pET21d
**Primers used for site-directed mutagenesis of Strep-linker-TEVsite-GRP94Δ72 in pET21d**			
GRP94 R116G	GCTTTAGACAAGATAGGGCTCATCTCC	GGAGATGAGCCCTATCTTGTCTAAAGC	pET21d
GRP94 K463E	CAAGGTGATTAGGAAGGAGCTTGTCC	GGACAAGCTCCTTCCTAATCACCTTG	pET21d
GRP94 K467E	GAAGAAGCTTGTCCGAGAAACTCTGG	CCAGAGTTTCTCGGACAAGCTTCTTC	pET21d
**Primers used for cloning the indicated protein constructs**			
BiP-NBD in pNIC28-Bsa4	TGAGCGGATAACAATTCCCCTCTAG	GCTCGAATTCGGATCCTCACAGGTCACCTGTATCTTGATCACCAGAGAGC	
Strep-GRP94 FL in pET21d	CGAGTGCGGCCGCAAGCTTTTACAATTCATCCTTCTCTGTAGATTCCTTTTCTG	CCCGTGAGACTCTTCAGTGTCATAAA	
Spot-HT2 in pET22b-SUMO	CCGGATCGCGTGCGCG	ACGGAGCTCGAATTCGGATCCTCAGCCGGCCAGCCC	
HT2-His6 in pET21a	CTAGCTAGCTCCGAAATCGGTACAGGCTTCC	CCGCTCGAGGCCGGCCAGCCCGG	

Supplementary Information on LC and MS settings

Overview

ACE project	Title
ACE_0791	Characterisation of BiP-GRP94-HT2 interaction
ACE_0925	Conformational plasticity of a BiP-GRP94 chaperone complex I
ACE_0953	Conformational plasticity of a BiP-GRP94 chaperone complex II

ACE_0791

File legend

ACE ID	Alt. sample ID	Organism	Organ/ cell line	Treatment/ experimental setup
ACE_0791_JB02		*mouse*	recombinant protein	Purified BiP and GRP94 were incubated in for 1h at 37 °C followed by 20 min RT DSBU crosslink. Ran SDS-PAGE and cut out Bands

LC_Settings

MS device	Orbitrap Fusion Lumos
LC device	Thermo Easy-nLC 1200
ion source	Thermo Nanospray Flex
**Analytical column**	Self-packed fused silica capillary without an integrated frit; CoAnn Technologies ICT36007515F-50-5
column diameter	Length (L_C) = 28 cm; ID = 75µm; OD = 360 µm; emitter 15 µm
stationary phase	Phenomenex Kinetex C18-XB core shell
particle diameter (d_p)	1.7 µm (core shell)
Pore size	100 Å
Column ID	AC145
Column oven	Sonation column oven PRSO-V2
Column oven temp.	50°C
**solvents**	A: 0.2% FA, 2% ACN, 97.8% H_2O<br>B: 0.2% FA, 80% ACN, 19.8 % H_2O
gradient	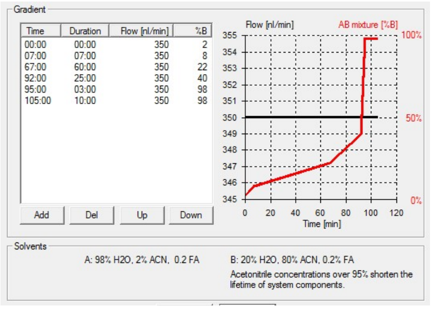

MS_settings

Project	MS	general	MS1	MS2	MS2	MS3	Comments; special settings
ACE_791	Lumos	Tune v3.3.2782.28<br>Gradient: 105 min	Analyzer: FT<br>Res.: 120000<br>SR: 375 - 1600<br>AGC: Standard<br>AcT: 50<br>RF: 30<br>SF: --<br>DDM: CT/5sec	Analyzer: IT<br>Res./ScR: 15000/-<br>SR: auto<br>AGC: Standard<br>AcT: 200 ms<br>CS: +3 to +7<br>IsM: Q<br>IsW: 2<br>Frag.: sHCD<br>NCE:27, 30, 33			classic orbitrap experiment: MS1 in Orbitrap at high resolution and data dependent MS2 in Iontrap at rapid scan rate. Dynamic exclusion enabled (exclude after n times=1; Exclusion duration (s)= 60; mass tolerance= ± 2 ppm)<br>intensity threshold: 20000

Note: **FT**= Fourier Transform (Orbitrap); **IT**= Iontrap; **Q**= Quadrupol; **Res.**= max. Resolution at 200 m/z (Lumos) or 400 m/z (Elite) [FWHM (full width at half maximum)]; **ScR**= scan rate for measurements in the IT; **SR**= scan range [m/z]; **AGC**= automatic gain control, max number of acquired ions per measurement; **AcT**= max. Ion acquisition time [ms]; **CS**= charge states used for fragmentation; **IsM**= Isolation mode (Q or IT), MS2 isolation and further is only done in IT; **IsW**= Isolation window [m/z], value followed by scan mode the isolation is based on (MS1, MS2 …) **Frag.**= Fragmentation method; **HCD**= Higher-energy collisional dissociation; **CID**= Collision-induced dissociation; **ETD**= Electron-transfer dissociation; **EThcD**= Electron-Transfer/Higher-Energy Collision Dissociation; **sHCD**= stepped HCD; **NCE**= normalized collision energy; **cycles**: number of MSn recorded or max cycle time; RF= RF Lens [%]; **SF**= Source Fragmentation [V]; **DDM**: Data dependent Mode (cycle time in seconds, CT/[s] or number of scans, NS); **NS**= Number of data dependent scans

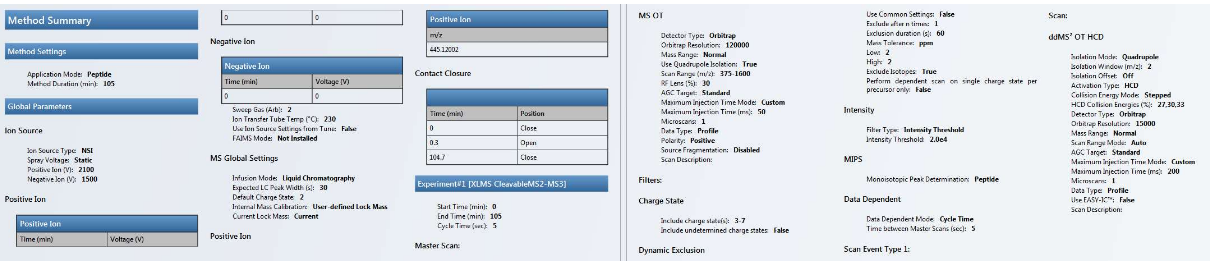

ACE_0925

File legend

ACE ID	Alt. sample ID	Organism	Organ/ cell line	Treatment/ experimental setup
ACE_0925 LB02	LBG5_F	*mouse*	recombinant protein	**0.6 % of DMSO** was mixed in **Buffer #A** and incubated for 10 minutes at room temperature. 50 µM of ATP and 4.0 µM BiP was added to the mix. This was then incubated for 1 hour at 37 degrees Celsius. 4.0 µM GRP94 FL was added to the reaction mix after the first 30 minutes. The reaction was then incubated at room temperature for 10 minutes. **0.5 mM of DSBU crosslinker** was added and incubated for 20 minutes at room temperature. The reaction was quenched with 20 mM Tris pH 8.0 and was incubated for 10 minutes at room temperature. The proteins are now in **Buffer #B**. 40 µl of the reaction was added to 4 µl of **Buffer #C** and incubated at 95 degrees Celsius for 5 minutes. The sample was loaded onto a 7.5 % SDS-PAGE gel and ran in **Buffer #D**. The gel was stained in **Buffer #E**. The band was cut (as depicted below in **Figure 1**).

Buffer #A	50 mM HEPES pH 7.5, 50 mM NaCl, 2 mM MgCl2
Buffer #B	50 mM HEPES pH 7.5, 50 mM NaCl, 2 mM MgCl2, 20 mM Tris pH 8.0
Buffer #C	200 mM Tris pH 6.8, 8% SDS, 40% glycerol, 4% beta-mercaptoethanol, bromophenol blue
Buffer #D	0.302 % Tris, 1.44 % Glycine, 0.1 % SDS
Buffer #E	0.25% Coomassie Brilliant Blue R-250, 45% ethanol, 10% acetic acid

LC_Settings

MS device	Orbitrap Fusion Lumos
LC device	Thermo Vanquish Neo
ion source	Thermo Nanospray Flex
**Analytical column**	Self-packed fused silica capillary with an integrated sintered frit; CoAnn Technologies ICT36007515F-50-5
column diameter	Length (L_C) = 28 cm; ID = 75µm; OD = 360 µm; emitter 15 µm
stationary phase	Phenomenex Kinetex C18-XB core shell
particle diameter (d_p)	1.7 µm
Pore size	120 Å
Column ID	AC156
Column oven	Sonation column oven PRSO-V2
Column oven temp.	50°C
**solvents**	A: 0.2% FA, 2% ACN, 97.8% H_2O<br>B: 0.2% FA, 80% ACN, 19.8 % H_2O
gradient	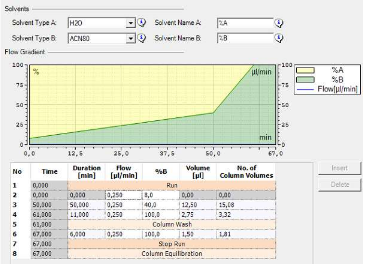

MS_settings

Project	MS	general	MS1	MS2	MS2	MS3	Comments; special settings
ACE_0925	Lumos	Tune v4.1.4244<br>Xcalibur v4.7.69.37<br>SII: 1.7.0.468<br>Gradient: 67	Analyzer: FT<br>Res.: 60000<br>SR: 380 - 1400<br>AGC: Standard<br>AGC abs.: 400000<br>AcT: Auto<br>RF: 30<br>SF: --<br>DDM: CT/3sec	Analyzer: FT<br>Res./ScR: 30000/-<br>SR: Auto<br>AGC: 200%<br>AGC abs.: 100000<br>AcT: 70 ms<br>CS: +3 to +8<br>IsM: Q<br>IsW: 1.6<br>Frag.: aHCD<br>NCE: 25, 30			classic orbitrap experiment: MS1 in Orbitrap at high resolution and data dependent MS2 also in Orbitrap high resolution. Dynamic exclusion enabled (exclude after n times=1; Exclusion duration (s)= 30; mass tolerance= ± 10ppm)<br><br>Intensity Threshold: 50000<br>Ion transfer Tube Temp: 270 °C<br>Ion Source Voltage: 2200 V

Note: **FT**= Fourier Transform (Orbitrap); **IT**= Iontrap; **Q**= Quadrupol; **Res.**= max. Resolution at 200 m/z (Lumos) or 400 m/z (Elite) [FWHM (full width at half maximum)]; **ScR**= scan rate for measurements in the IT; **SR**= scan range [m/z]; **AGC**= automatic gain control, max number of acquired ions per measurement; **AcT**= max. Ion acquisition time [ms]; **CS**= charge states used for fragmentation; **IsM**= Isolation mode (Q or IT), MS2 isolation and further is only done in IT; **IsW**= Isolation window [m/z], value followed by scan mode the isolation is based on (MS1, MS2 …) **Frag.**= Fragmentation method; **HCD**= Higher-energy collisional dissociation; **CID**= Collision-induced dissociation; **ETD**= Electron-transfer dissociation**; EThcD=** Electron-Transfer/Higher-Energy Collision Dissociation; **sHCD**= stepped HCD**; NCE**= normalized collision energy; **cycles**: number of MSn recorded or max cycle time; RF= RF Lens [%]; **SF**= Source Fragmentation [V]; **DDM**: Data dependent Mode (cycle time in seconds, CT/[s] or number of scans, NS); **NS**= Number of data dependent scans

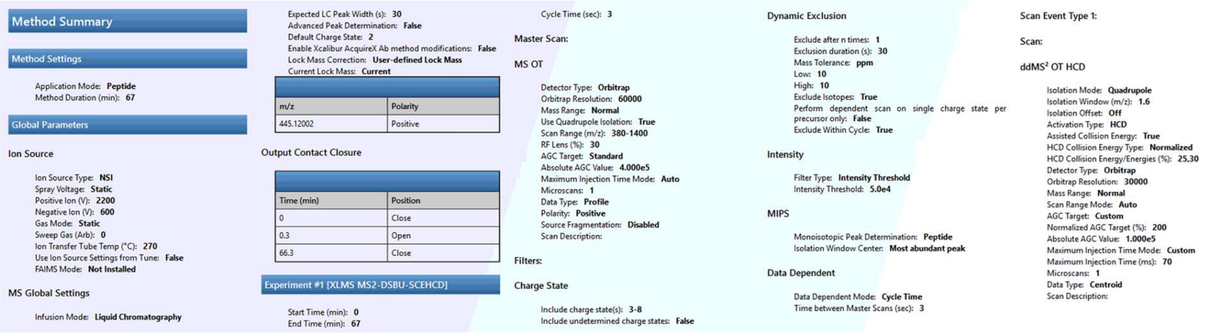

Method Summary

Method Settings

Application Mode: **Peptide**
Method Duration (min): **67**

Global Parameters

Ion Source

Ion Source Type: **NSI**
Spray Voltage: **Static**
Positive Ion (V): **2200**
Negative Ion (V): **600**
Gas Mode: **Static**
Sweep Gas (Arb): **0**
Ion Transfer Tube Temp (°C): **270**
Use Ion Source Settings from Tune: **False**
FAIMS Mode: **Not Installed**

MS Global Settings

Infusion Mode: **Liquid Chromatography**
Expected LC Peak Width (s): **30**
Advanced Peak Determination: **False**
Default Charge State: **2**
Enable Xcalibur AcquireX Ab method modifications: **False**
Lock Mass Correction: **User-defined Lock Mass**
Current Lock Mass: **Current**

m/z	Polarity
445.12002	Positive

Output Contact Closure

Time (min)	Position
0	Close
0.3	Open
66.3	Close

Experiment #1 [XLMS MS2-DSBU-SCEHCD]

Start Time (min): **0**
End Time (min): **67**
Cycle Time (sec): **3**

Master Scan:

MS OT

Detector Type: **Orbitrap**
Orbitrap Resolution: **60000**
Mass Range: **Normal**
Use Quadrupole Isolation: **True**
Scan Range (m/z): **380-1400**
RF Lens (%): **30**
AGC Target: **Standard**
Absolute AGC Value: **4.000e5**
Maximum Injection Time Mode: **Auto**
Microscans: **1**
Data Type: **Profile**
Polarity: **Positive**
Source Fragmentation: **Disabled**
Scan Description:

Filters:

Charge State

Include charge state(s): **3-8**
Include undetermined charge states: **False**

Dynamic Exclusion

Exclude after n times: **1**
Exclusion duration (s): **30**
Mass Tolerance: **ppm**
Low: **10**
High: **10**
Exclude Isotopes: **True**
Perform dependent scan on single charge state per precursor only: **False**
Exclude Within Cycle: **True**

Intensity

Filter Type: **Intensity Threshold**
Intensity Threshold: **5.0e4**

MIPS

Monoisotopic Peak Determination: **Peptide**
Isolation Window Center: **Most abundant peak**

Data Dependent

Data Dependent Mode: **Cycle Time**
Time between Master Scans (sec): **3**

Scan Event Type 1:

Scan:

$ddMS^2$ OT HCD

Isolation Mode: **Quadrupole**
Isolation Window (m/z): **1.6**
Isolation Offset: **Off**
Activation Type: **HCD**
Assisted Collision Energy: **True**
HCD Collision Energy Type: **Normalized**
HCD Collision Energy/Energies (%): **25,30**
Detector Type: **Orbitrap**
Orbitrap Resolution: **30000**
Mass Range: **Normal**
Scan Range Mode: **Auto**
AGC Target: **Custom**
Normalized AGC Target (%): **200**
Absolute AGC Value: **1.000e5**
Maximum Injection Time Mode: **Custom**
Maximum Injection Time (ms): **70**
Microscans: **1**
Data Type: **Centroid**
Scan Description:

ACE_0953

File legend

ACE ID	Alt. sample ID	Organism	Organ/ cell line	Treatment/ experimental setup
ACE_0953_LB03	LB148_G2_S6_LB	*mouse*	recombinant protein	4.0 µM GRP94 was mixed with **100 mM PU-WS13** in **Buffer #A** for 30 minutes at 37 °C. 50 µM of ATP and 4.0 µM BiP-NBD were added to the reaction and incubated for an additional 30 minutes at 37 °C (total reaction time = 1 hour at 37 °C). The reaction was then incubated at room temperature for 5 minutes. **0.5 mM of DSBU crosslinker** was added and incubated for 20 minutes at room temperature. The reaction was quenched with 20 mM Tris pH 8.0 and was incubated for 10 minutes at room temperature. The proteins at the step are in **Buffer #B**. 30 µl of the reaction was added to 10 µl of **Buffer #C** and incubated at 95 degrees Celsius for 5 minutes. 6.7 µl of the sample before and after crosslinking were loaded onto a 4-15% pre-cast SDS-PAGE gel and ran in **Buffer #D** for 3 hours. The gel was stained in **Buffer #E**. The band was cut (as depicted below in the **Figure below**).
ACE_0953_LB05	LB155_Gel			4.0 µM GRP94 was mixed with **100 mM PU-WS13** in **Buffer #A** for 30 minutes at 37 °C. 50 µM of ATP and 4.0 µM BiP were added to the reaction and incubated for an additional 30 minutes at 37 °C (total reaction time = 1 hour at 37 °C). The reaction was then incubated at room temperature for 5 minutes. **0.5 mM of DSBU crosslinker** was added and incubated for 20 minutes at room temperature. The reaction was quenched with 20 mM Tris pH 8.0 and was incubated for 10 minutes at room temperature. The proteins at the step are in **Buffer #B**. 30 µl of the reaction was added to 10 µl of **Buffer #C** and incubated at 95 degrees Celsius for 5 minutes. 20 µl of the sample before and after crosslinking were loaded onto a 4-15% pre-cast SDS-PAGE gel and ran in **Buffer #D** for 3 hours. The gel was stained in **Buffer #E**. The band was cut (as depicted below in the **Figure below**).

Buffer #A	50 mM HEPES pH 7.5, 50 mM NaCl, 2 mM MgCl2
Buffer #B	50 mM HEPES pH 7.5, 50 mM NaCl, 2 mM MgCl2, 20 mM Tris pH 8.0
Buffer #C	200 mM Tris pH 6.8, 8% SDS, 40% glycerol, 4% beta-mercaptoethanol, bromophenol blue
Buffer #D	0.302 % Tris, 1.44 % Glycine, 0.1 % SDS
Buffer #E	0.25% Coomassie Brilliant Blue R-250, 45% ethanol, 10% acetic acid

LC_Settings

MS device	Orbitrap Fusion Lumos
LC device	Thermo Vanquish Neo
ion source	Thermo Nanospray Flex
**Analytical column**	Self-packed fused silica capillary with an integrated sintered frit; CoAnn Technologies ICT36007515F-50-5
column diameter	Length (L_C) = 32 cm; ID = 75µm; OD = 360 µm; emitter 15 µm
stationary phase	Phenomenex Kinetex C18-XB core shell
particle diameter (d_p)	1.7 µm
Pore size	120 Å
Column ID	AC160
Column oven	Sonation column oven PRSO-V2
Column oven temp.	50°C
**solvents**	A: 0.2% FA, 2% ACN, 97.8% H_2O<br>B: 0.2% FA, 80% ACN, 19.8 % H_2O
gradient	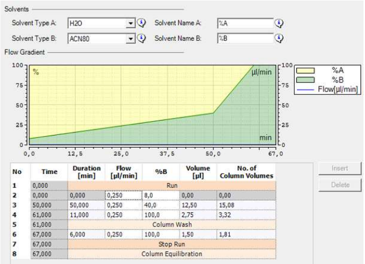

No	Time	Duration [min]	Flow [µl/min]	%B	Volume [µl]	No. of Column Volumes
1	0,000	Run				
2	0,000	0,000	0,250	8,0	0,00	0,00
3	50,000	50,000	0,250	40,0	12,50	15,08
4	61,000	11,000	0,250	100,0	2,75	3,32
5	61,000	Column Wash				
6	67,000	6,000	0,250	100,0	1,50	1,81
7	67,000	Stop Run				
8	67,000	Column Equilibration				

MS_settings

Project	MS	general	MS1	MS2	MS2	MS3	Comments; special settings
ACE_0953	Lumos	Tune v4.1.4244<br>Xcalibur v4.7.69.37<br>SII: 1.7.0.468<br>Gradient: 67	Analyzer: FT<br>Res.: 60000<br>SR: 380 - 1400<br>AGC: Standard<br>AGC abs.: 400000<br>AcT: Auto<br>RF: 30<br>SF: --<br>DDM: CT/3sec	Analyzer: FT<br>Res./ScR: 30000/-<br>SR: Auto<br>AGC: 200%<br>AGC abs.: 100000<br>AcT: 70 ms<br>CS: +3 to +8<br>IsM: Q<br>IsW: 1.6<br>Frag.: aHCD<br>NCE: 25, 30			classic orbitrap experiment: MS1 in Orbitrap at high resolution and data dependent MS2 also in Orbitrap high resolution. Dynamic exclusion enabled (exclude after n times=1; Exclusion duration (s)= 30; mass tolerance= ± 10ppm)<br><br>Intensity Threshold: 50000<br>Ion transfer Tube Temp: 270 °C<br>Ion Source Voltage: 2200 V

Note: **FT**= Fourier Transform (Orbitrap); **IT**= Iontrap; **Q**= Quadrupol; **Res.**= max. Resolution at 200 m/z (Lumos) or 400 m/z (Elite) [FWHM (full width at half maximum)]; **ScR**= scan rate for measurements in the IT; **SR**= scan range [m/z]; **AGC**= automatic gain control, max number of acquired ions per measurement; **AcT**= max. Ion acquisition time [ms]; **CS**= charge states used for fragmentation; **IsM**= Isolation mode (Q or IT), MS2 isolation and further is only done in IT; **IsW**= Isolation window [m/z], value followed by scan mode the isolation is based on (MS1, MS2 …) **Frag.**= Fragmentation method; **HCD**= Higher-energy collisional dissociation; **CID**= Collision-induced dissociation; **ETD**= Electron-transfer dissociation; **EThcD=** Electron-Transfer/Higher-Energy Collision Dissociation; **sHCD**= stepped HCD**; NCE**= normalized collision energy; **cycles**: number of MSn recorded or max cycle time; RF= RF Lens [%]; **SF**= Source Fragmentation [V]; **DDM**: Data dependent Mode (cycle time in seconds, CT/[s] or number of scans, NS); **NS**= Number of data dependent scans

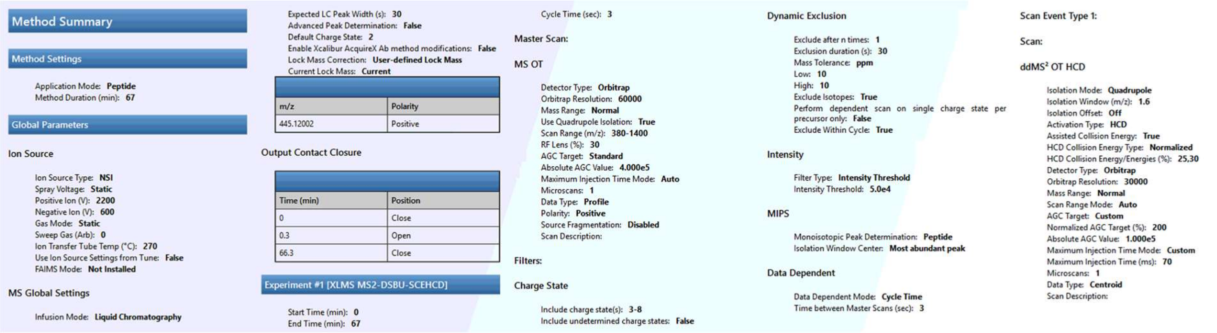

Supplement: Supplementary file 1 — Supplementary Table 5 and information on LC–MS settings. [file 41594_2025_1619_MOESM1_ESM.pdf]
