# Supplementary material for: Conformational plasticity of a BiP–GRP94 chaperone complex
Source: Nat Struct Mol Biol. 2025 Jul 14;32(10):1947–58. doi: 10.1038/s41594-025-01619-0 (PMC12527940; doi:10.1038/s41594-025-01619-0)
Supplement: Supplementary file 2 — Reporting Summary [file 41594_2025_1619_MOESM2_ESM.pdf]

nature portfolio

Corresponding author(s): Doris Hellerschmied

Last updated by author(s): May 14, 2025

Reporting Summary

Nature Portfolio wishes to improve the reproducibility of the work that we publish. This form provides structure for consistency and transparency in reporting. For further information on Nature Portfolio policies, see our Editorial Policies and the Editorial Policy Checklist.

Statistics

For all statistical analyses, confirm that the following items are present in the figure legend, table legend, main text, or Methods section.

n/a	Confirmed	
☐	☒	The exact sample size (*n*) for each experimental group/condition, given as a discrete number and unit of measurement
☐	☒	A statement on whether measurements were taken from distinct samples or whether the same sample was measured repeatedly
☒	☐	The statistical test(s) used AND whether they are one- or two-sided<br>*Only common tests should be described solely by name; describe more complex techniques in the Methods section.*
☒	☐	A description of all covariates tested
☒	☐	A description of any assumptions or corrections, such as tests of normality and adjustment for multiple comparisons
☐	☒	A full description of the statistical parameters including central tendency (e.g. means) or other basic estimates (e.g. regression coefficient) AND variation (e.g. standard deviation) or associated estimates of uncertainty (e.g. confidence intervals)
☒	☐	For null hypothesis testing, the test statistic (e.g. *F*, *t*, *r*) with confidence intervals, effect sizes, degrees of freedom and *P* value noted<br>*Give P values as exact values whenever suitable.*
☒	☐	For Bayesian analysis, information on the choice of priors and Markov chain Monte Carlo settings
☒	☐	For hierarchical and complex designs, identification of the appropriate level for tests and full reporting of outcomes
☒	☐	Estimates of effect sizes (e.g. Cohen's *d*, Pearson's *r*), indicating how they were calculated

Our web collection on statistics for biologists contains articles on many of the points above.

Software and code

Policy information about availability of computer code

Data collection	MS data were collected on an Orbitrap LUMOS instrument (Thermo) coupled to an EASY-nLC 1200 ultra-performance liquid chromatography (UPLC) system (Thermo). Negative-stain EM data were collected on a Talos L120C microscope (Thermo Fisher) with a CMOS camera (Ceta 16M).
Data analysis	Negative-stain EM data was processed in CryoSPARC v4, using CTFFIND4 for CTF estimation and Blob picker for particle picking. XL-MS data were processed with ProteoWizard (version: 3.0.23018-60066e9), Xisearch (version: 1.7.6.7), and xiFDR (version 2.1.5.5). For molecular modelling and analysis of structural data SWISS-MODEL web server (accessed 13.10.2023), UCSF Chimera (version 1.17.3), UCSF Chimera X (version 1.5), Rosetta (release 314, 2022.11+release.512e589) , and PyMOL 2.5.7 were used. For image quantification ImageLab 6.1 (BioRad) and Prism 10.1.2 (Graphpad) were used.

For manuscripts utilizing custom algorithms or software that are central to the research but not yet described in published literature, software must be made available to editors and reviewers. We strongly encourage code deposition in a community repository (e.g. GitHub). See the Nature Portfolio guidelines for submitting code & software for further information.

Data

Policy information about availability of data

All manuscripts must include a data availability statement. This statement should provide the following information, where applicable:

- Accession codes, unique identifiers, or web links for publicly available datasets
- A description of any restrictions on data availability
- For clinical datasets or third party data, please ensure that the statement adheres to our policy

The mass spectrometry proteomics data have been deposited to the ProteomeXchange Consortium via the PRIDE partner repository (https://www.ebi.ac.uk/pride/archive/) with the dataset identifier PXD059917.
Negative-stain EM maps have been deposited in the Electron Microscopy Data Bank (EMDB) under the accession numbers EMD-19600 and EMD-19601. The following publicly accessible PDB models were used for molecular modeling and data analysis in this work: 2KHO, 2O1V, 5E84, 5E85, 5ULS, 6DWS, 7KW7, 8TF0.

Research involving human participants, their data, or biological material

Policy information about studies with human participants or human data. See also policy information about sex, gender (identity/presentation), and sexual orientation and race, ethnicity and racism.

Reporting on sex and gender	n/a
Reporting on race, ethnicity, or other socially relevant groupings	n/a
Population characteristics	n/a
Recruitment	n/a
Ethics oversight	n/a

Note that full information on the approval of the study protocol must also be provided in the manuscript.

Field-specific reporting

Please select the one below that is the best fit for your research. If you are not sure, read the appropriate sections before making your selection.

☒ Life sciences ☐ Behavioural & social sciences ☐ Ecological, evolutionary & environmental sciences

For a reference copy of the document with all sections, see nature.com/documents/nr-reporting-summary-flat.pdf

Life sciences study design

All studies must disclose on these points even when the disclosure is negative.

Sample size	Sample sizes for biochemical experiments were chosen based on common practice in the field to account for experimental and measurement variation and to ensure reproducible results: *) Analytical size exclusion chromatography runs, limited proteolysis, and crosslinking experiments were repeated at least twice and gave reproducible and consitent results. *) For quantifications of pull-down and crosslinking experiments three independent biological replicates were performed (as reported in the figure legends).<br>Particle numbers for single-particle negative-stain EM are described in detail in Extended Data Figure, including a flow chart indicating the number and percentages of particles that were used for each corresponding 3D reconstruction.
Data exclusions	No data was excluded.
Replication	The number of replications is stated in the figure legends and/or in the Statistics and Reproducibility statement.
Randomization	Randomization was not applicable, since experiments did not involve humans or animals. Moreover, the scientist performing the biochemical experiments needed to be aware of which proteins have to be added to the tubes.
Blinding	No blinding was performed, since no subjective analysis was performed.

Reporting for specific materials, systems and methods

We require information from authors about some types of materials, experimental systems and methods used in many studies. Here, indicate whether each material, system or method listed is relevant to your study. If you are not sure if a list item applies to your research, read the appropriate section before selecting a response.

Materials & experimental systems

n/a	Involved in the study
☐	☒ Antibodies
☐	☒ Eukaryotic cell lines
☒	☐ Palaeontology and archaeology
☒	☐ Animals and other organisms
☒	☐ Clinical data
☒	☐ Dual use research of concern
☒	☐ Plants

Methods

n/a	Involved in the study
☒	☐ ChIP-seq
☒	☐ Flow cytometry
☒	☐ MRI-based neuroimaging

Antibodies

Antibodies used

anti-His5 (Santa Cruz Biotechnologies, SC-8036), anti-Strep (Qiagen, 34850), anti-BiP C50B12 (Cell Signaling, 3177), anti-GRP94 (Proteintech, 14700-1-AP), anti-HA-tag C29F4 (Cell Signaling 3724), anti-HaloTag (Promega, G9211), Goat anti-mouse HRP-coupled secondary antibody (Invitrogen, 31444), and Goat anti-rabbit HRP-coupled secondary antibody (Invitrogen, 31460)

Validation

The anti-His5, anti-Strep, anti-HaloTag, and anti-HA antibodies were used to detect tags on purified proteins or proteins overexpressed in HEK293 cells. No band was detected when the corresponding tagged protein was omitted from the sample, validating the use of these antibodies in our experimental setup. Specifically, Extended Data Figure 2c and 6b show that the anti-Strep antibody recognizes Strep-tagged GRP94. No band was detected for samples not containing any Strep-GRP94 protein (see Extended Data Figure 2c – lanes containing BiPNBD only). Extended Data Figure 6b also shows that the anti-HaloTag antibody specifically detects a single band at the expected molecular weight for HaloTag2 and does not react with BiP or GRP94, which are also present in the input sample. The anti-His5 antibody detects the BiPNBD at the expected molecular weight as demonstrated in Extended Data Figure 2b.
The anti-HA tag antibody was used to detect B4GT-HA-EGFP-HT2, which is expressed upon Doxycycline induction in HEK293 cells. As demonstrated in Hellerschmied et al MBoC 2019, Figure 1D, the antibody specifically recognizes the HA-tagged protein upon addition of Doxycycline.
Anti-BiP and anti-GRP94 antibodies were approved for use in Western blot experiments by their respective manufacturers. ProteinTech provides Western blot proof of function for the anti-GRP94 antibody in HEK293 cells (used in this work) and a GRP94 knock-down validation (in prostate cancer cells) is for example provided in this publication 10.1515/biol-2019-0043
https://www.ptglab.com/products/HSP90B1-Antibody-14700-1-AP.htm
Cell signaling provides proof of function for Western blot and showcases over 900 publications using the anti-BiP (3177) antibody, as listed on the following website
https://www.cellsignal.com/products/primary-antibodies/bip-c50b12-rabbit-mab/3177

Eukaryotic cell lines

Policy information about cell lines and Sex and Gender in Research

Cell line source(s)

Stable HEK293 Flp-in Trex cells (Thermo Fisher Scientific) expressing B4GT-HA-EGFP-HT2 were previously generated and described in Serebrenik et al., 2018.

Authentication

The HEK293 Flp-in Trex cell line used was not authenticated.

Mycoplasma contamination

The cell line tested negative for mycoplasma contamination using the VenorGeM OneStep mycoplasma detection kit.

Commonly misidentified lines
(See ICLAC register)

No commonly misidentified cell line was used in this work.

Plants

Seed stocks

Report on the source of all seed stocks or other plant material used. If applicable, state the seed stock centre and catalogue number. If plant specimens were collected from the field, describe the collection location, date and sampling procedures.

Novel plant genotypes

Describe the methods by which all novel plant genotypes were produced. This includes those generated by transgenic approaches, gene editing, chemical/radiation-based mutagenesis and hybridization. For transgenic lines, describe the transformation method, the number of independent lines analyzed and the generation upon which experiments were performed. For gene-edited lines, describe the editor used, the endogenous sequence targeted for editing, the targeting guide RNA sequence (if applicable) and how the editor was applied.

Authentication

Describe any authentication procedures for each seed stock used or novel genotype generated. Describe any experiments used to assess the effect of a mutation and, where applicable, how potential secondary effects (e.g. second site T-DNA insertions, mosiacism, off-target gene editing) were examined.
