# Supplementary material for: Conformational plasticity of a BiP–GRP94 chaperone complex
Source: Nat Struct Mol Biol. 2025 Jul 14;32(10):1947–58. doi: 10.1038/s41594-025-01619-0 (PMC12527940; doi:10.1038/s41594-025-01619-0)
Supplement: Supplementary file 10 — Unprocessed gels for Figs. 1 and 3–5 and Extended Data Figs. 1, 2, 5 and 6. [file 41594_2025_1619_MOESM10_ESM.pdf]

Data Source Figures

Figure 1

(b) and (c) 2

(d) and (e) 3

Figure 2

(b) and (d) 4

(e) and (f) 5

Figure 4

(f) 6

Figure 5

(c) 7

(d) and (f) 8

Extended Data Source Figures

Figure 1

(a) and (b) 9

Figure 2

(a) 10-11

(b) 12

(c) 13-15

(d) 16

Figure 5 17-19

Figure 6 20

Data Source Figure 1b and 1c

b

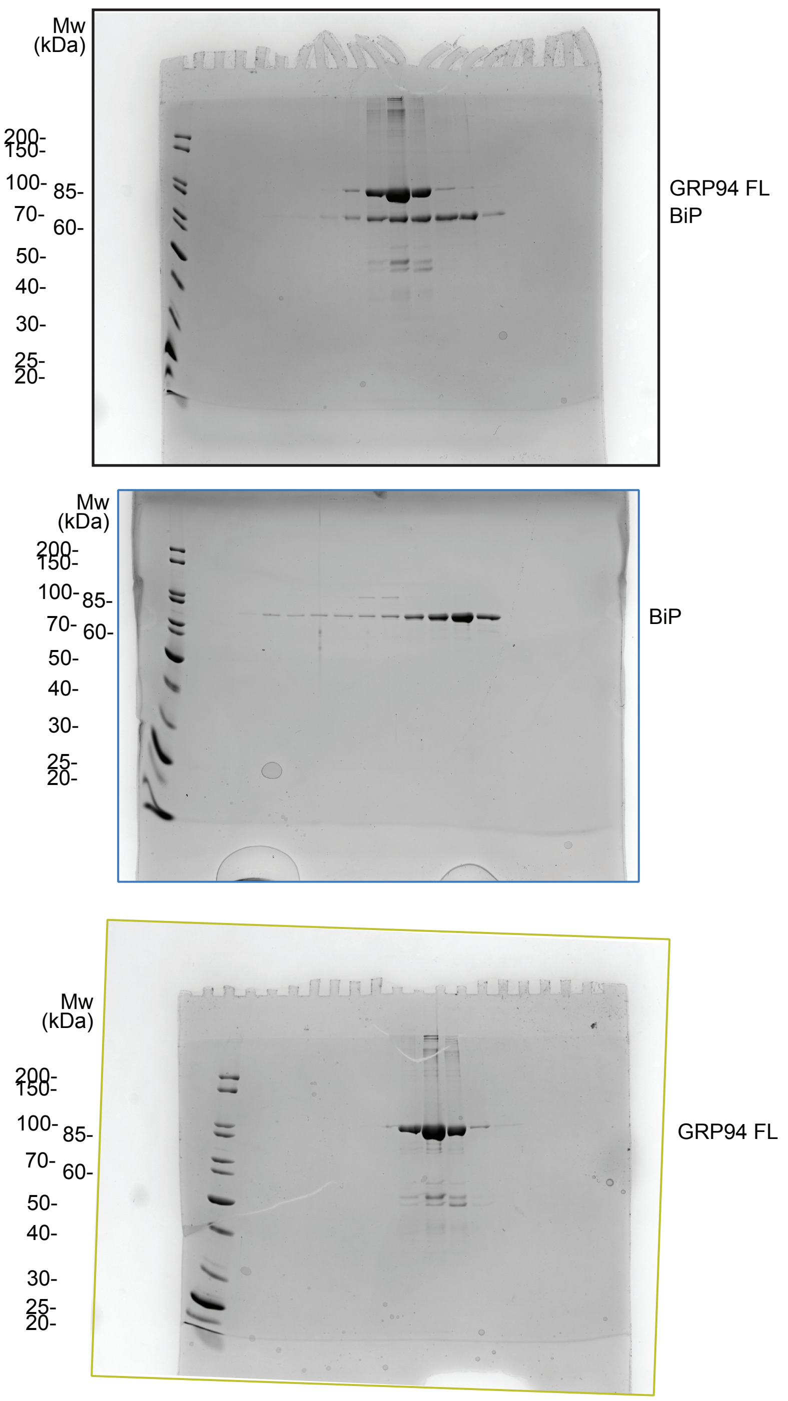

c

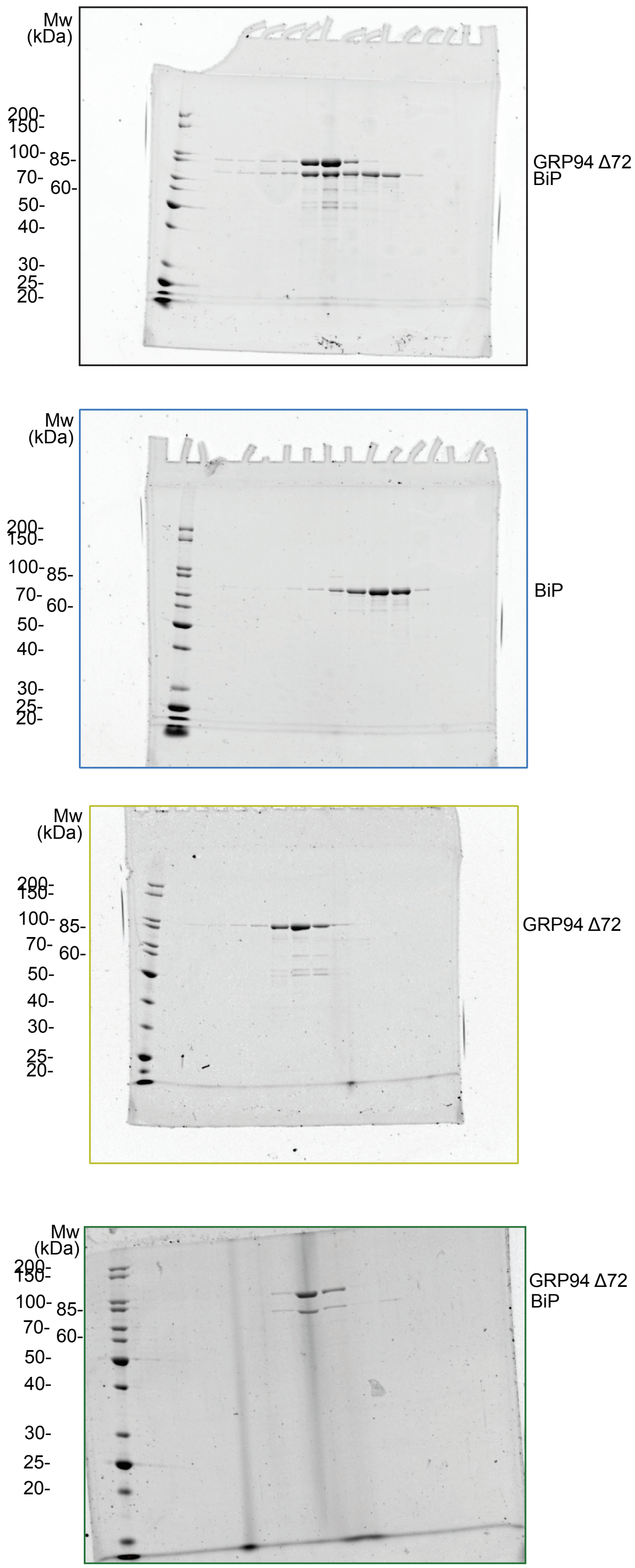

Data Source Figure 1d and 1e

d

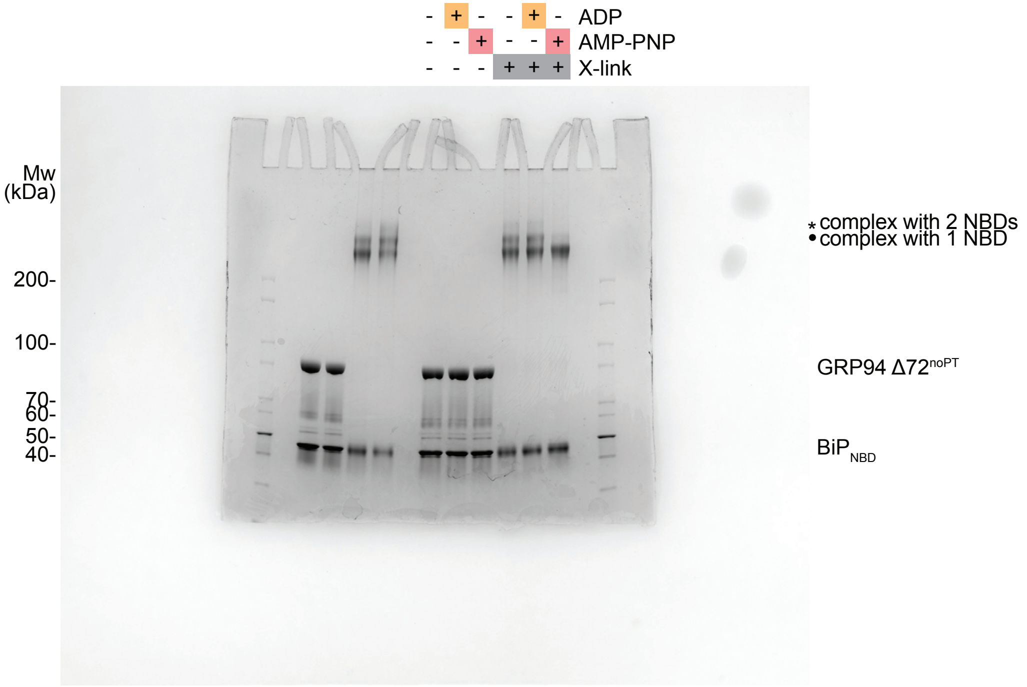

e

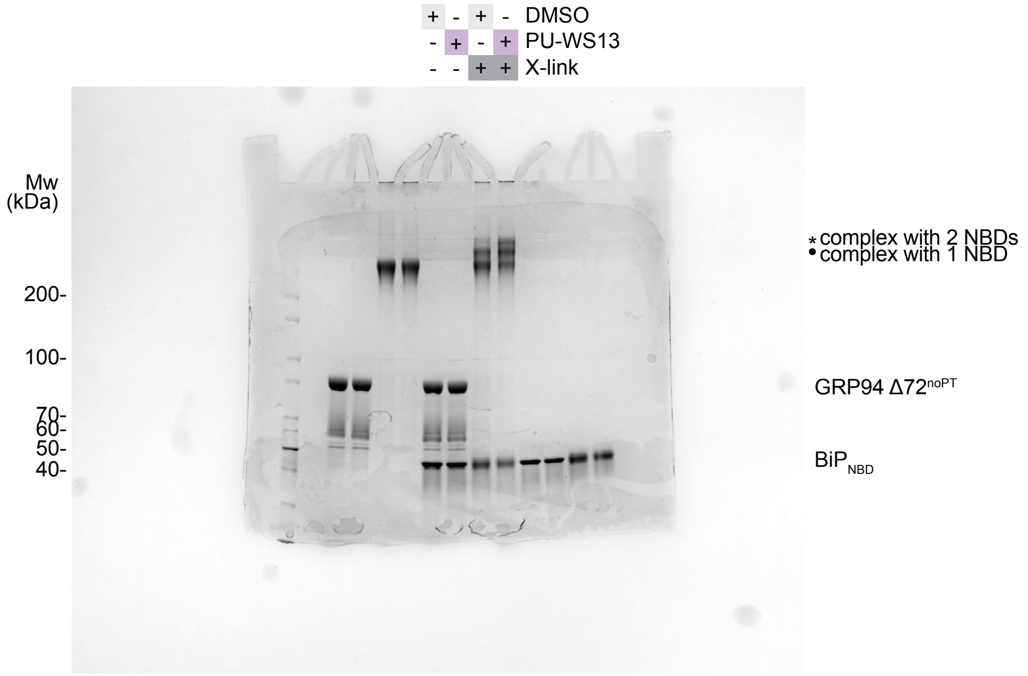

Data Source Figure 2b and 2d

b

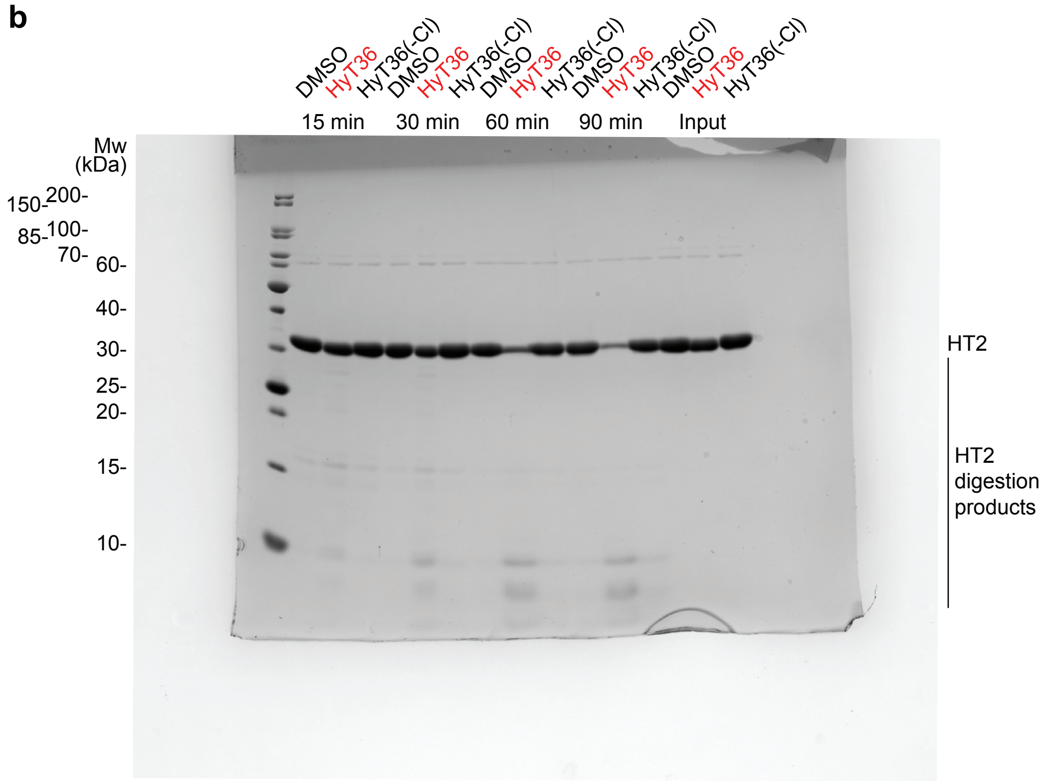

d

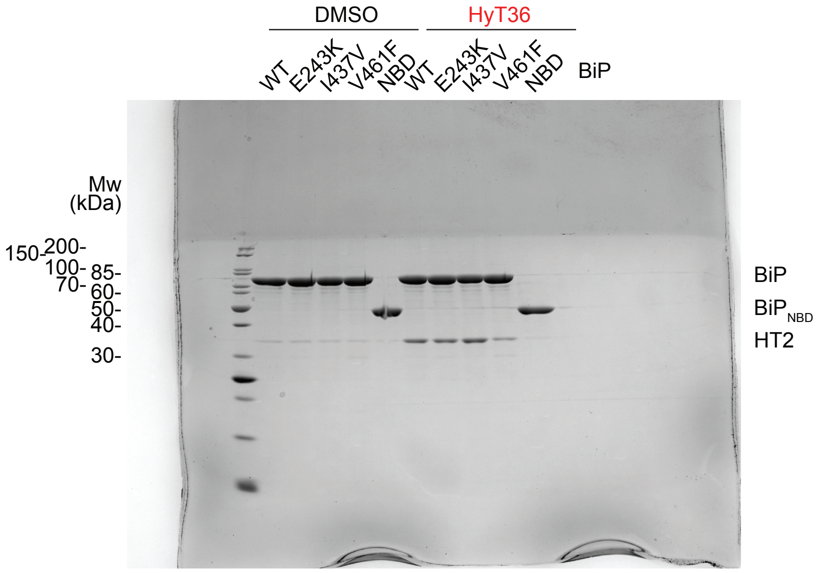

elution

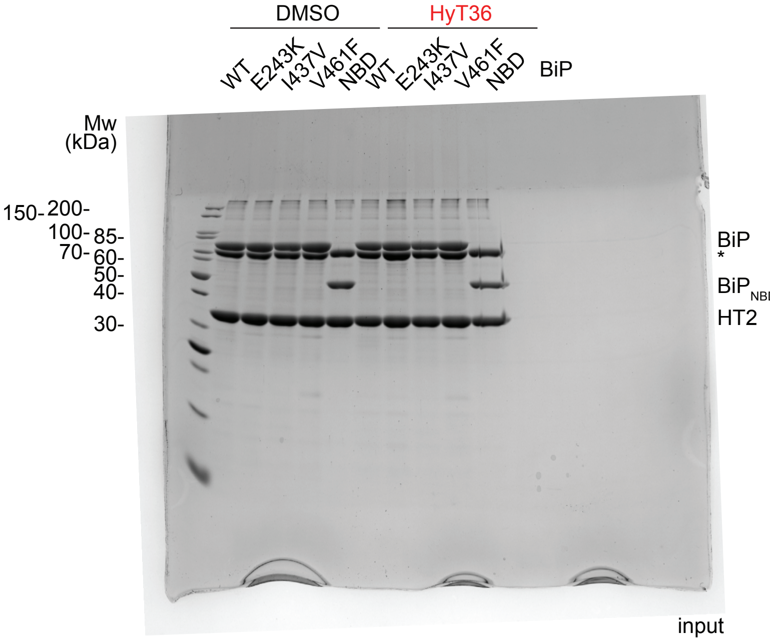

input

Data Source Figure 2e and 2f

e

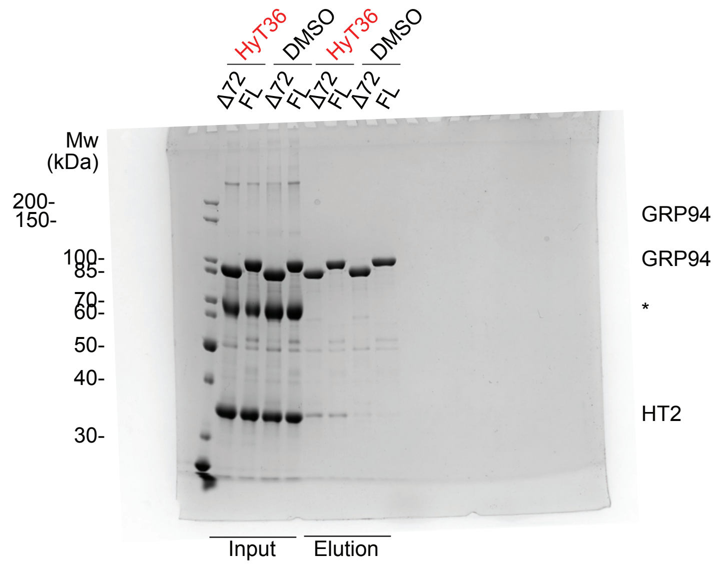

f

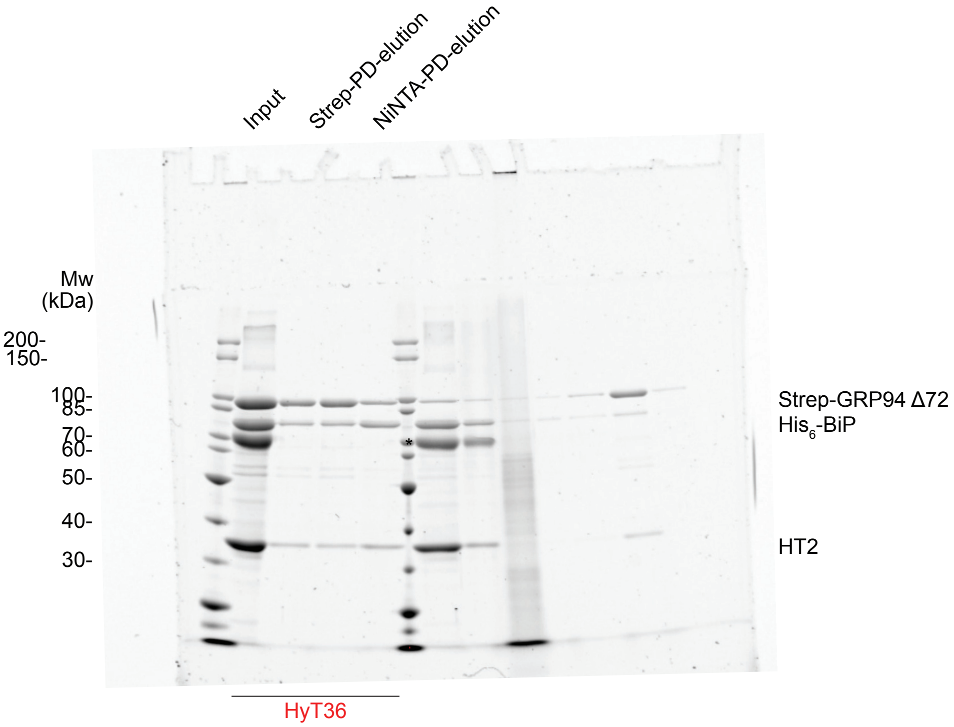

Data Source Figure 4f

f

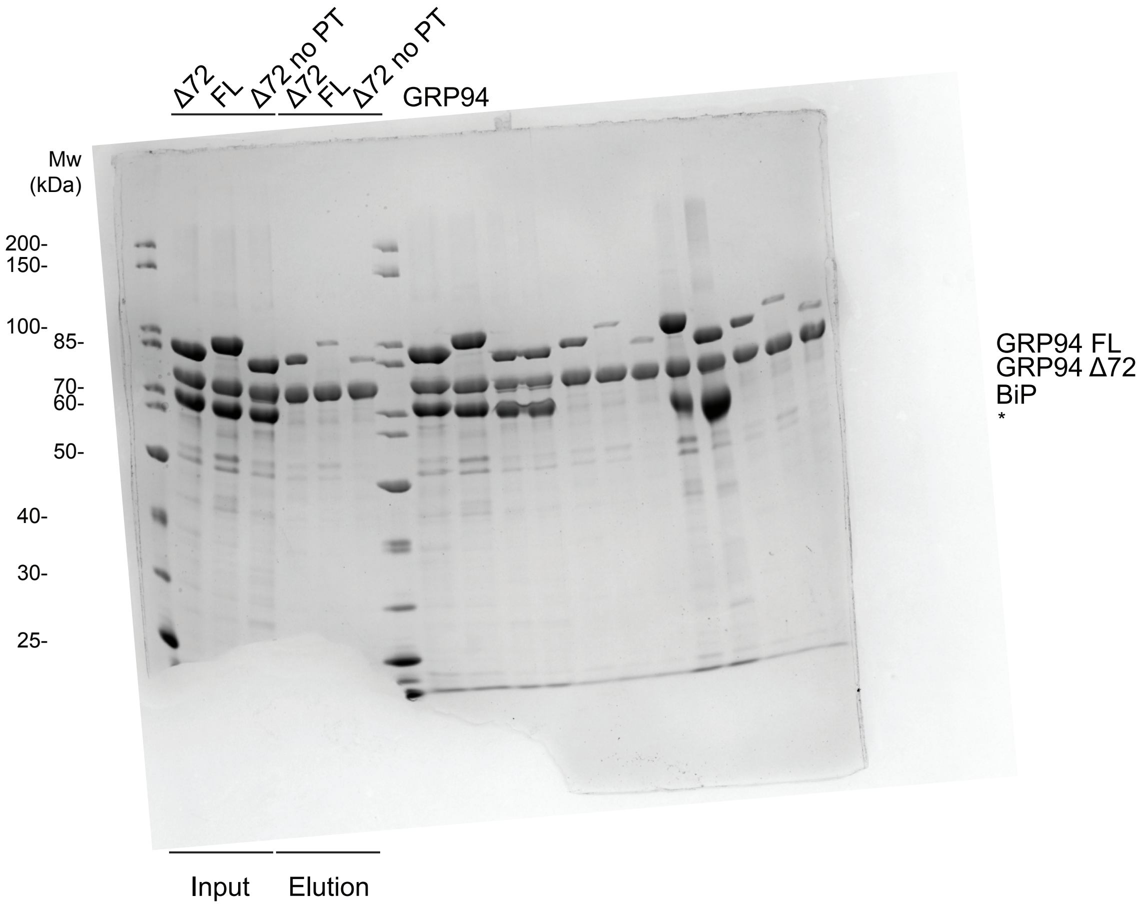

Data Source Figure 5c

c

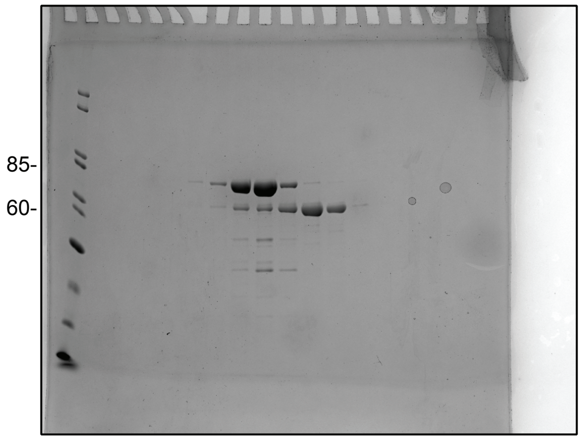

GRP94 Δ72
BiP E243K

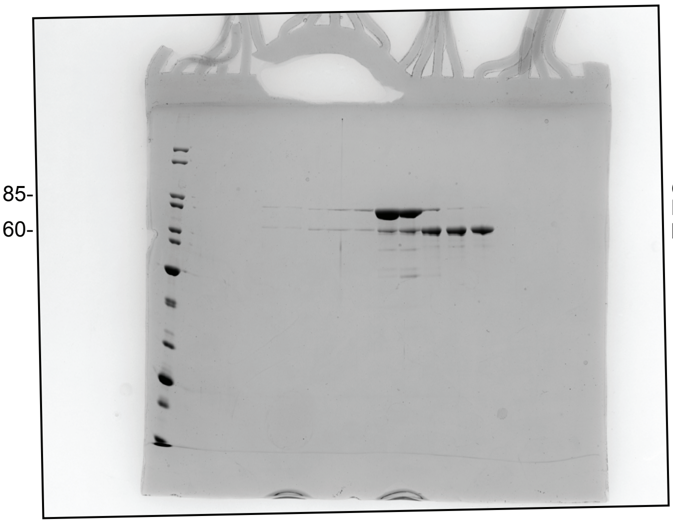

GRP94 Δ72
K463E
BiP

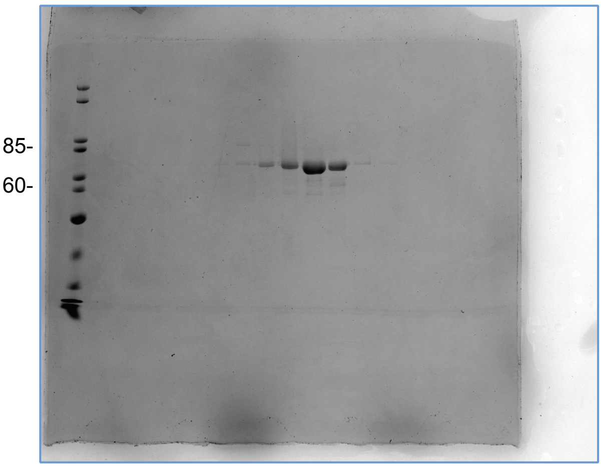

BiP E243K

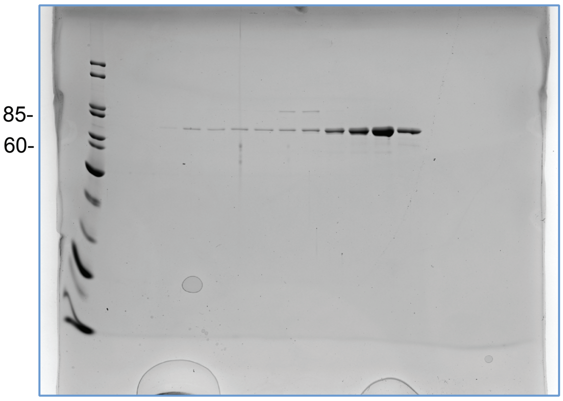

BiP

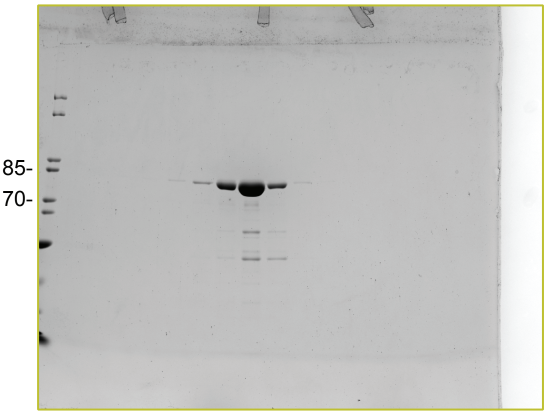

GRP94 Δ72

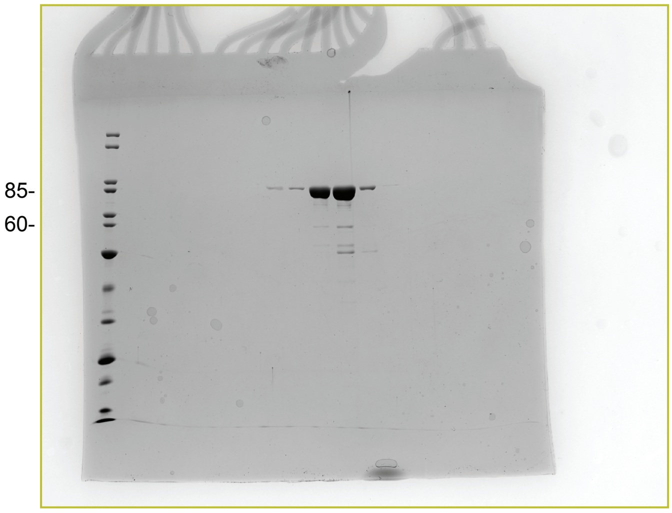

GRP94 Δ72
K463E

Data Source Figure 5d and 5f

d

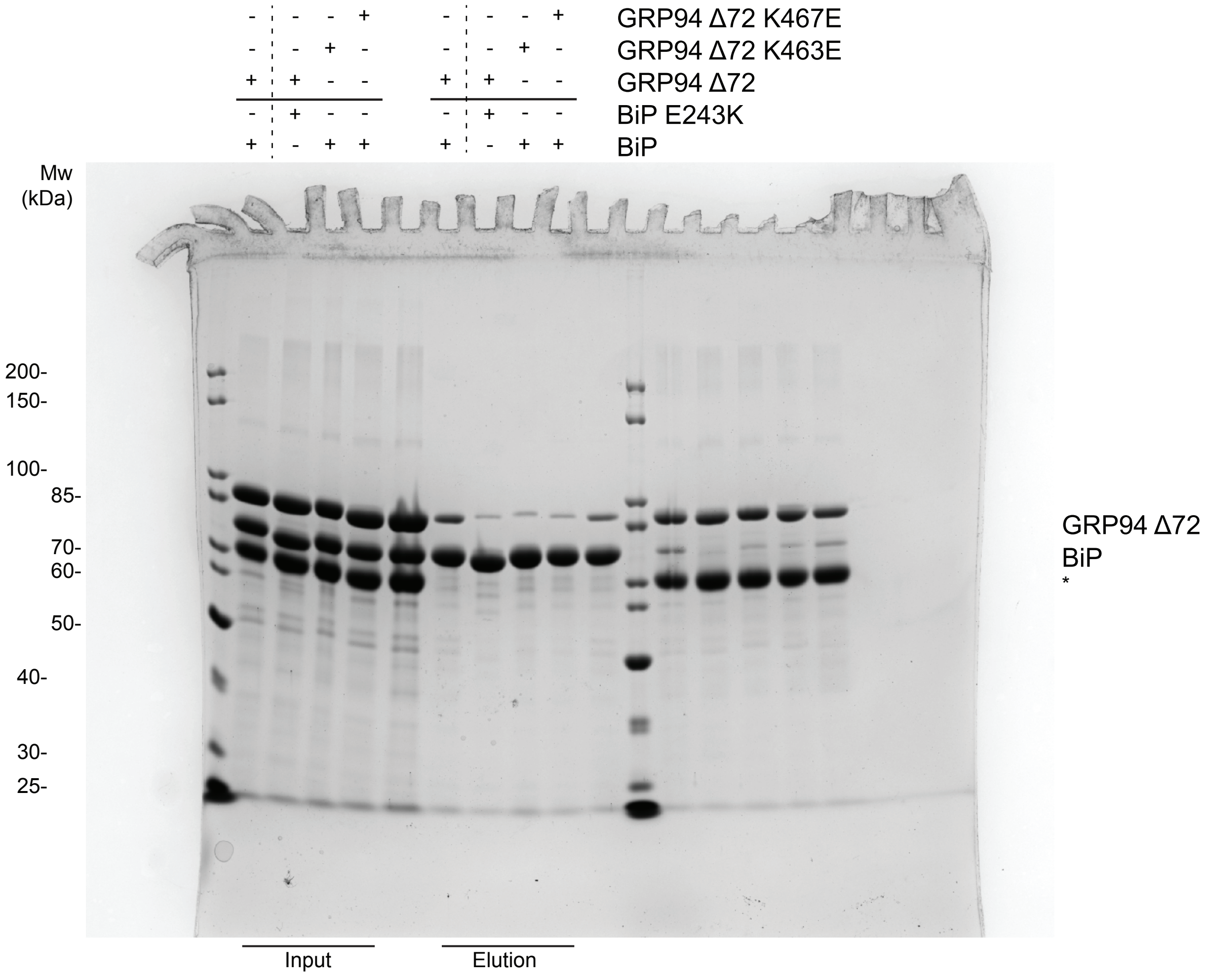

f

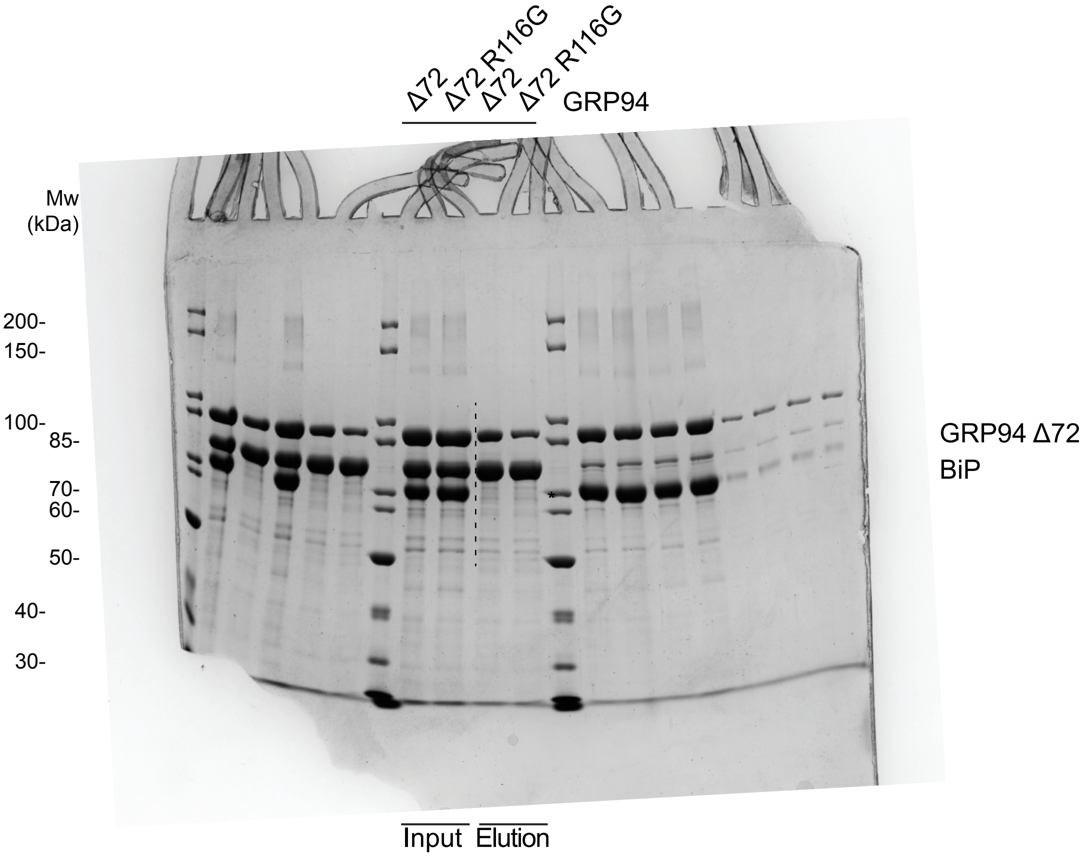

Data Source Extended Data Figure 1a and 1b

a

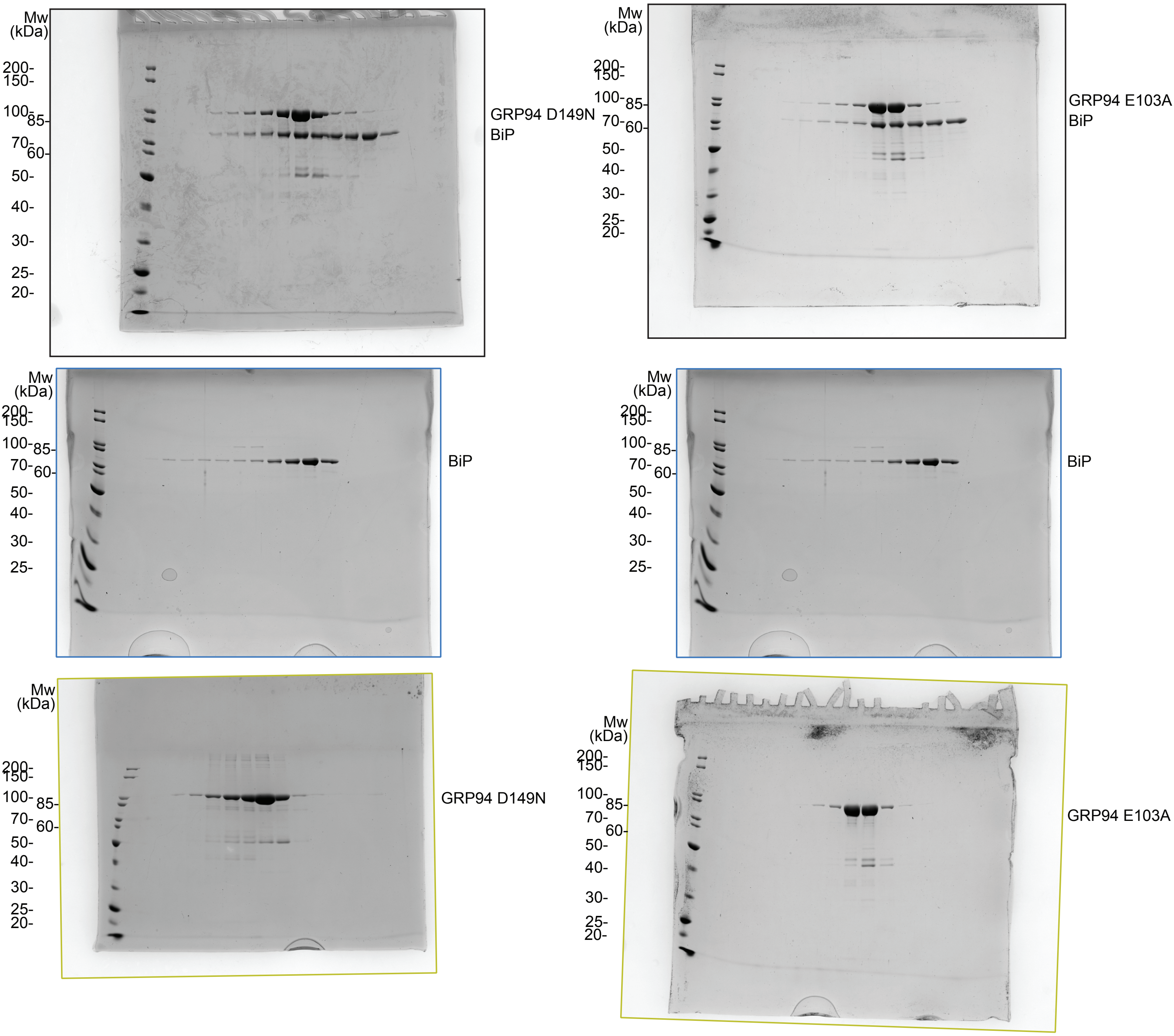

b

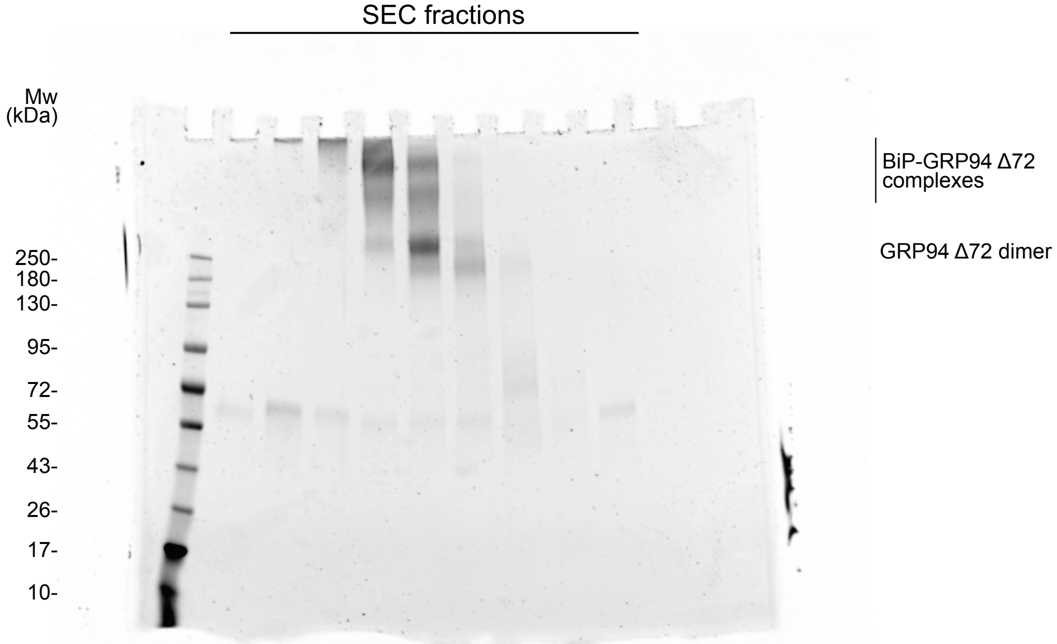

Data Source Extended Data Figure 2a

a

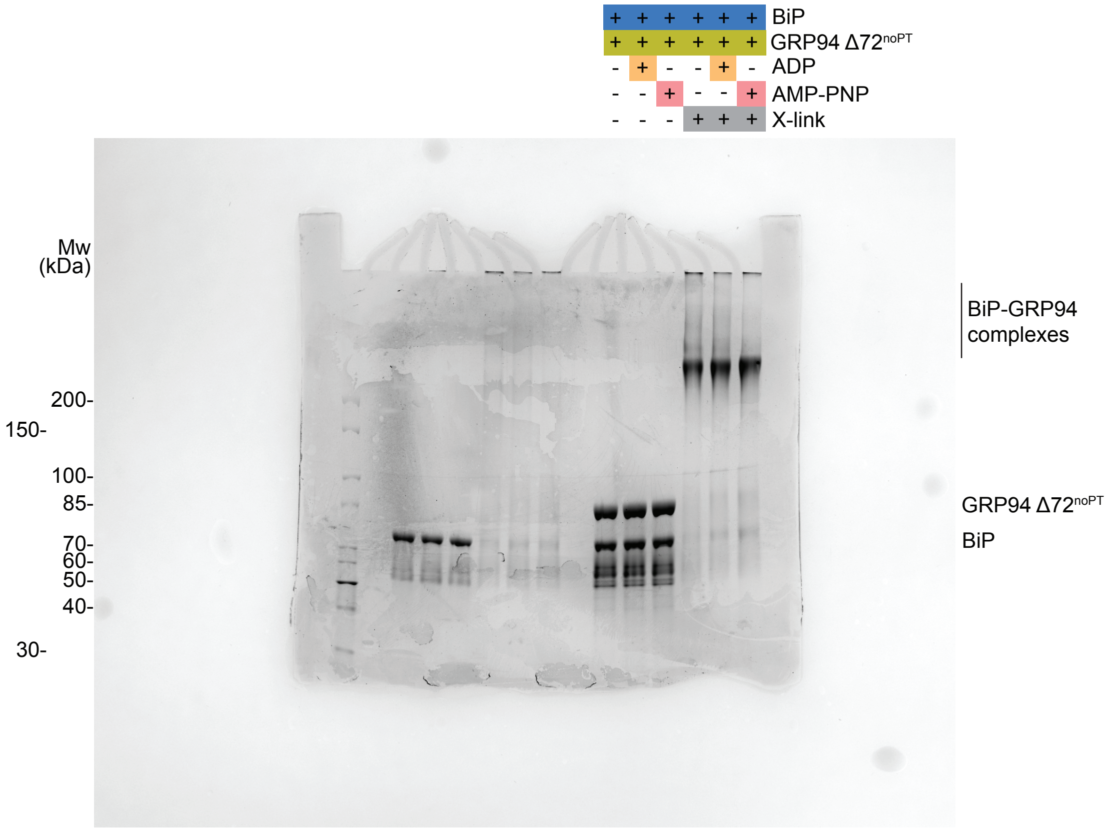

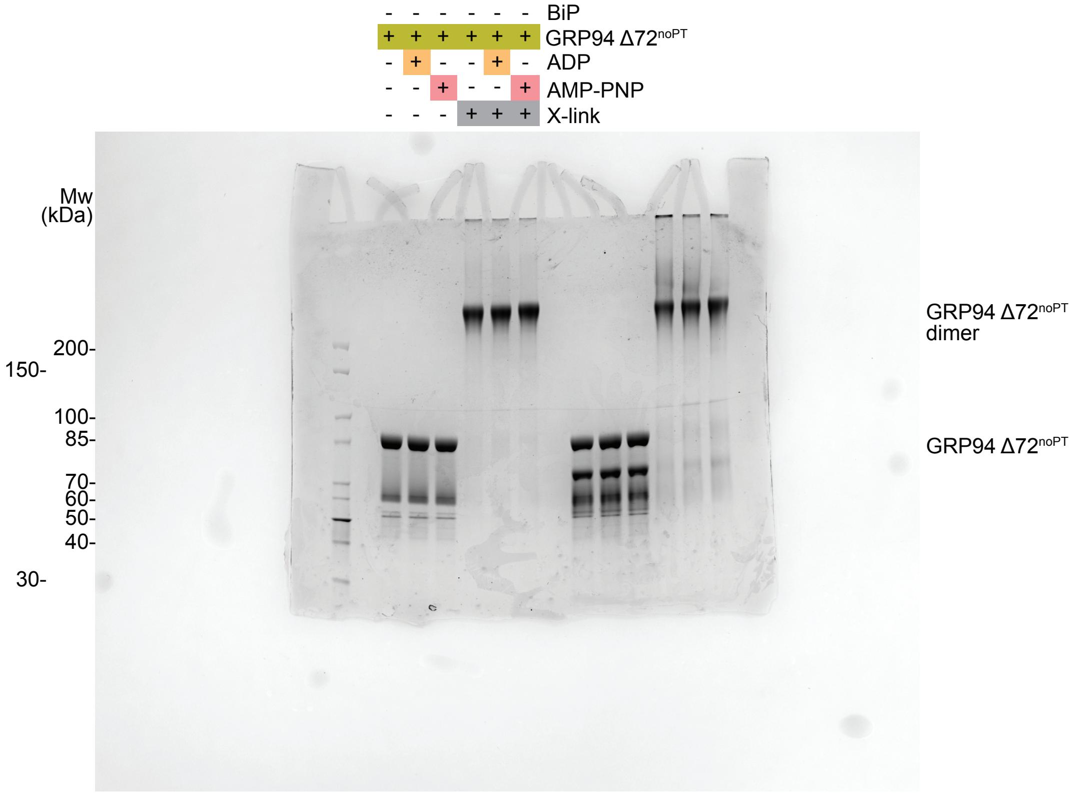

Data Source Extended Data Figure 2a cont.

a

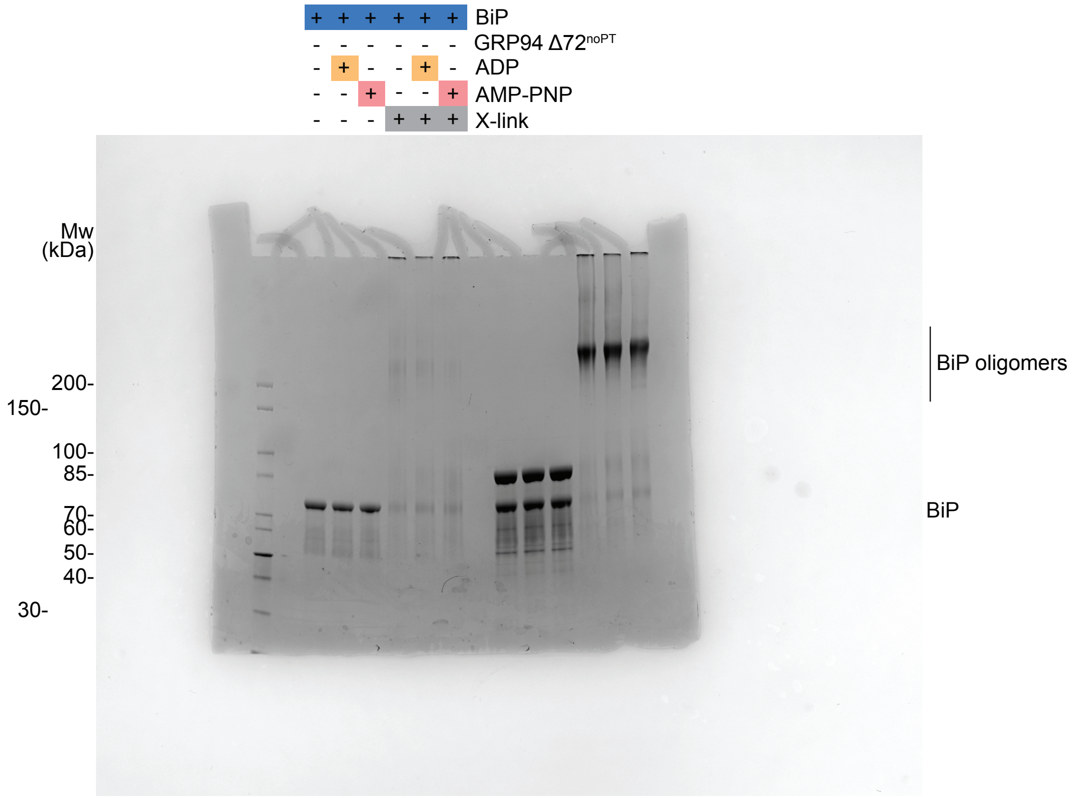

Data Source Extended Data Figure 2b

b

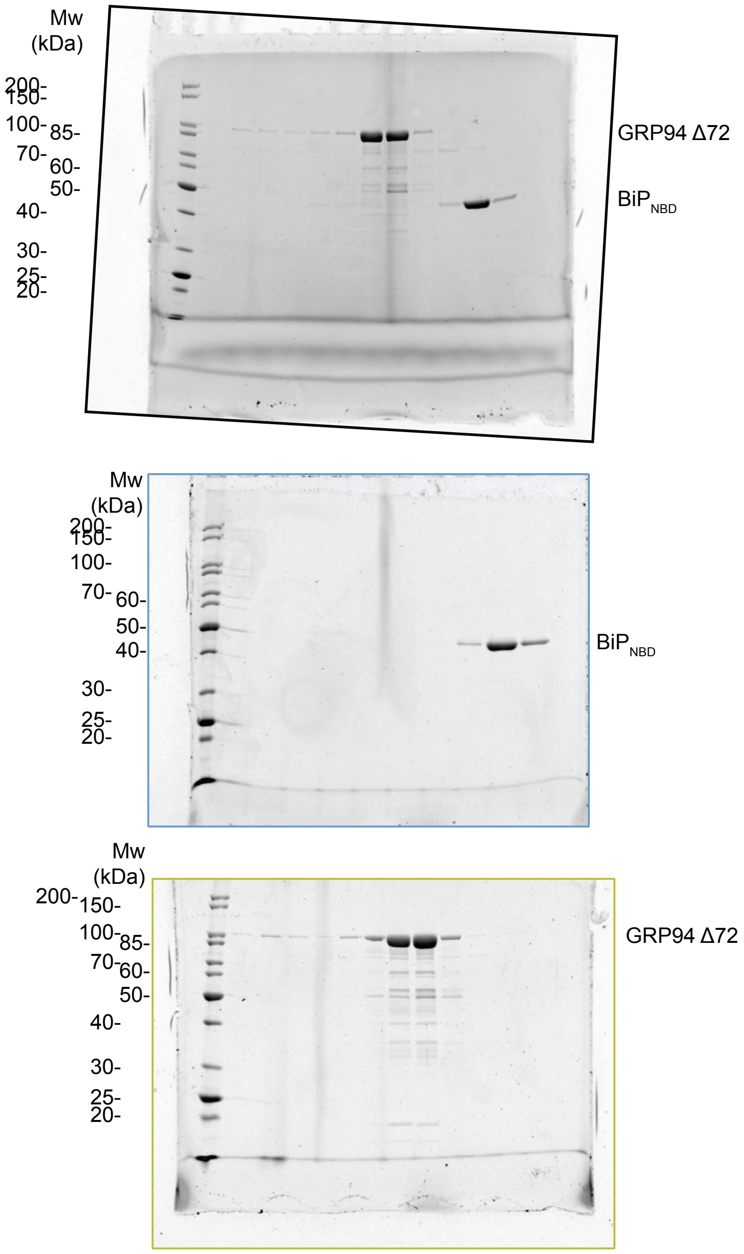

Data Source Extended Data Figure 2c

c

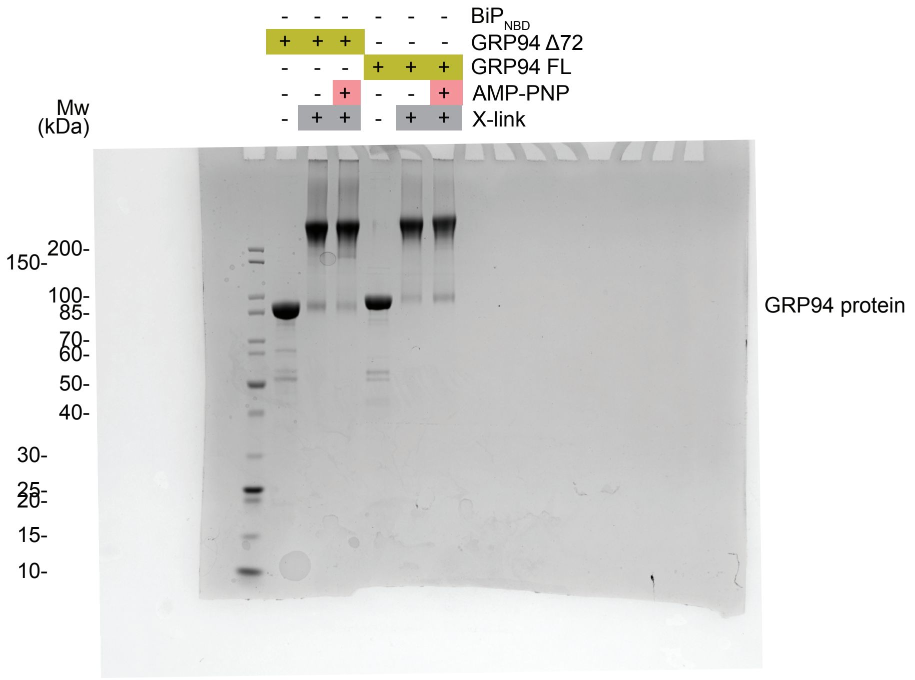

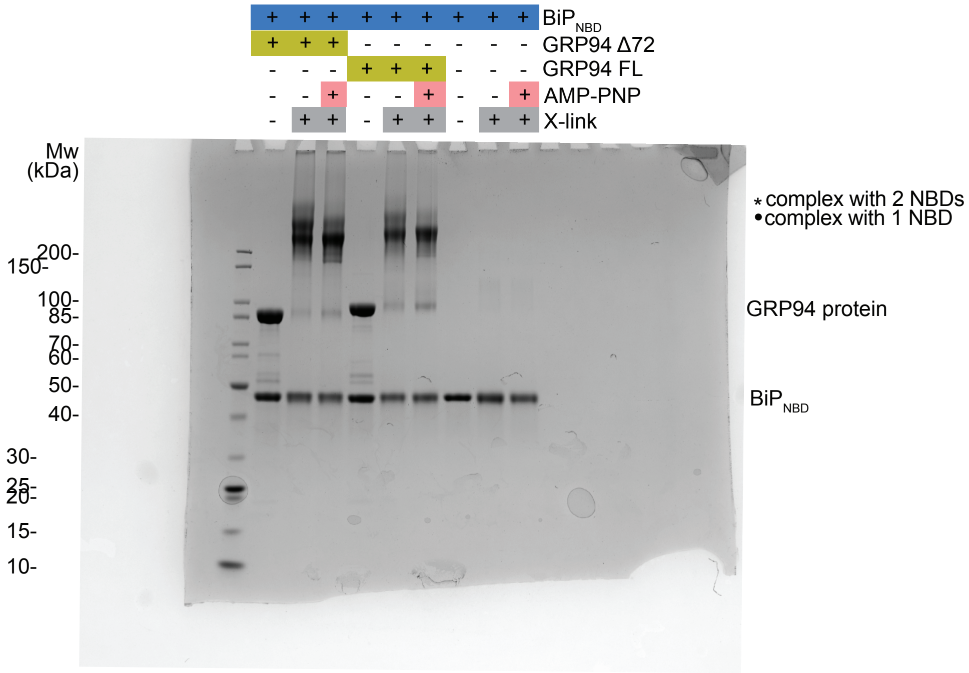

Data Source Extended Data Figure 2c cont.1

c

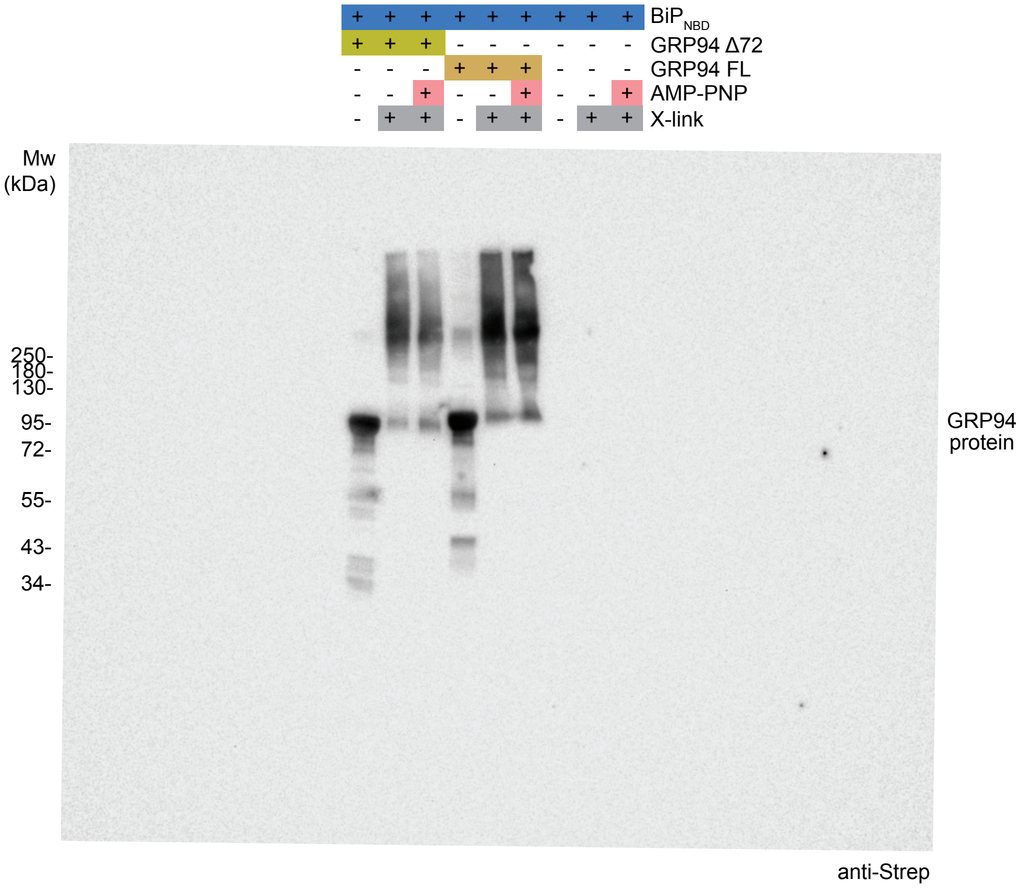

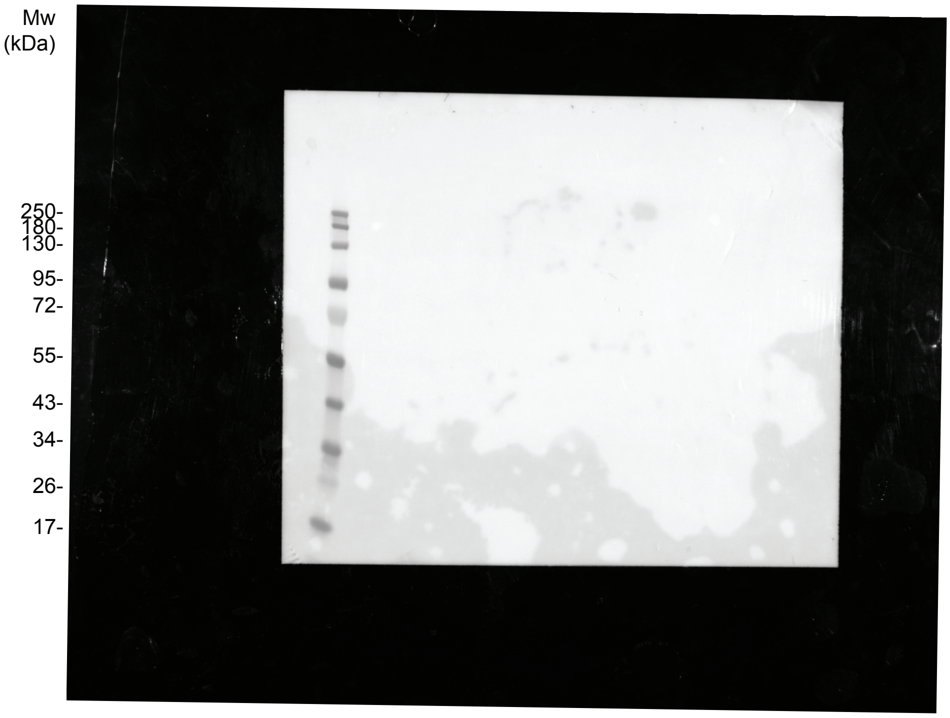

Data Source Extended Data Figure 2c cont.2

c

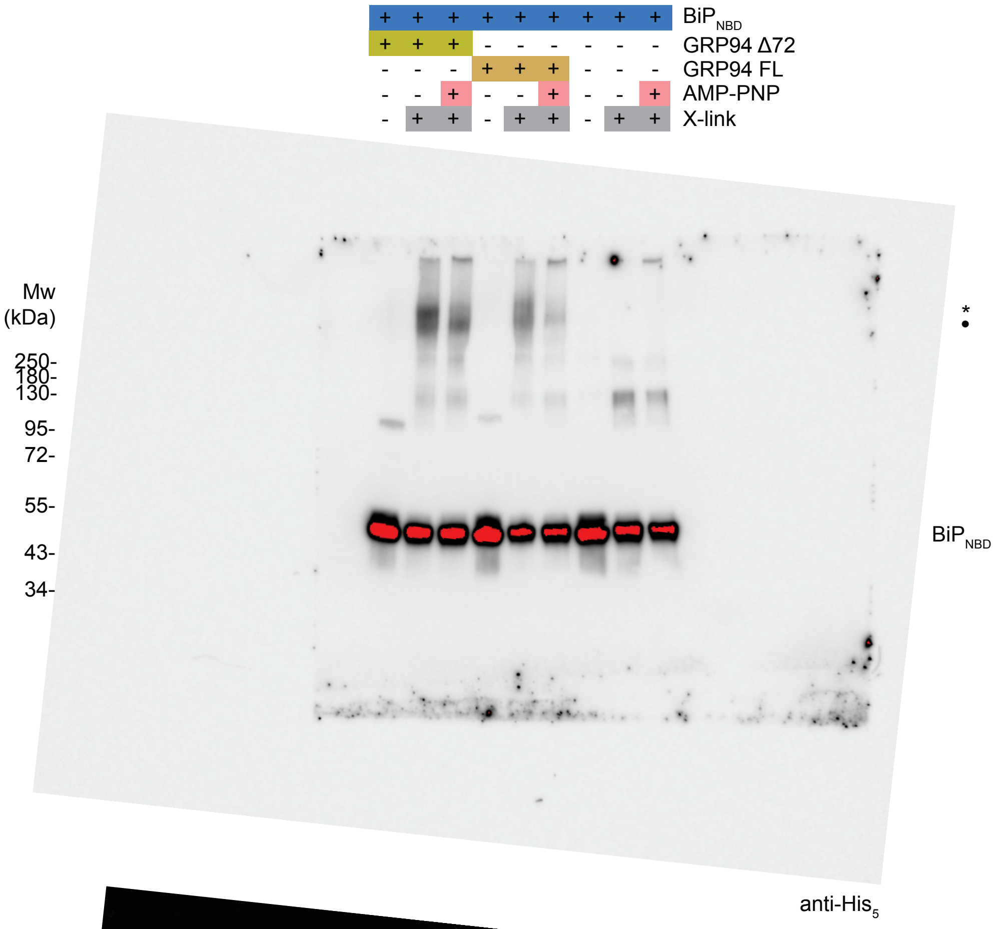

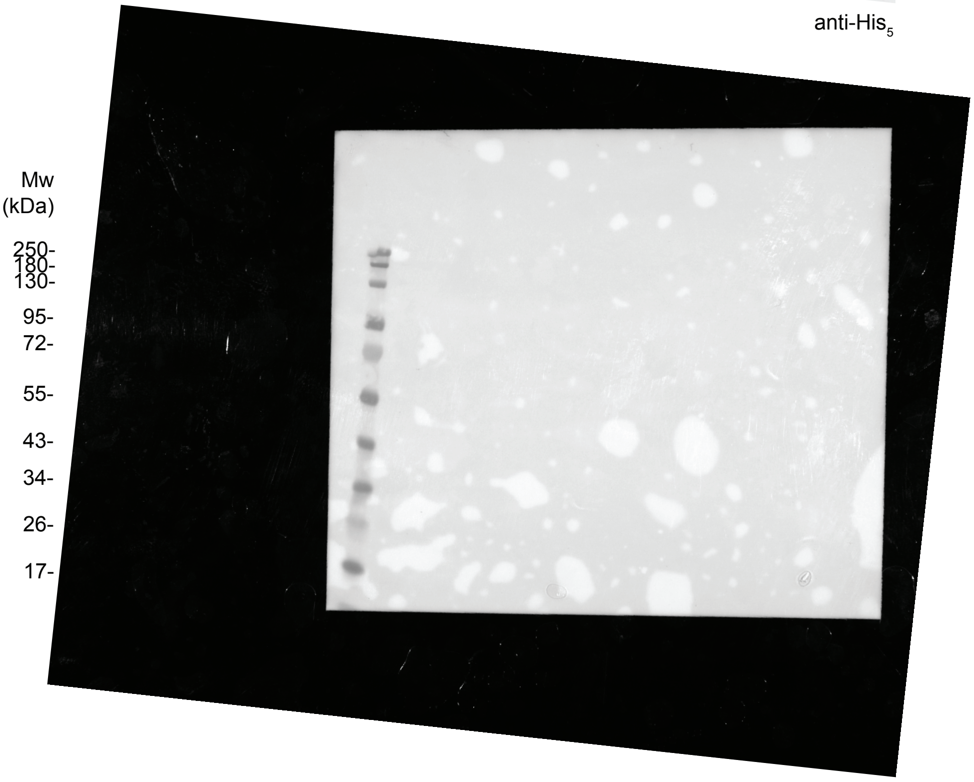

Data Source Extended Data Figure 2d

d

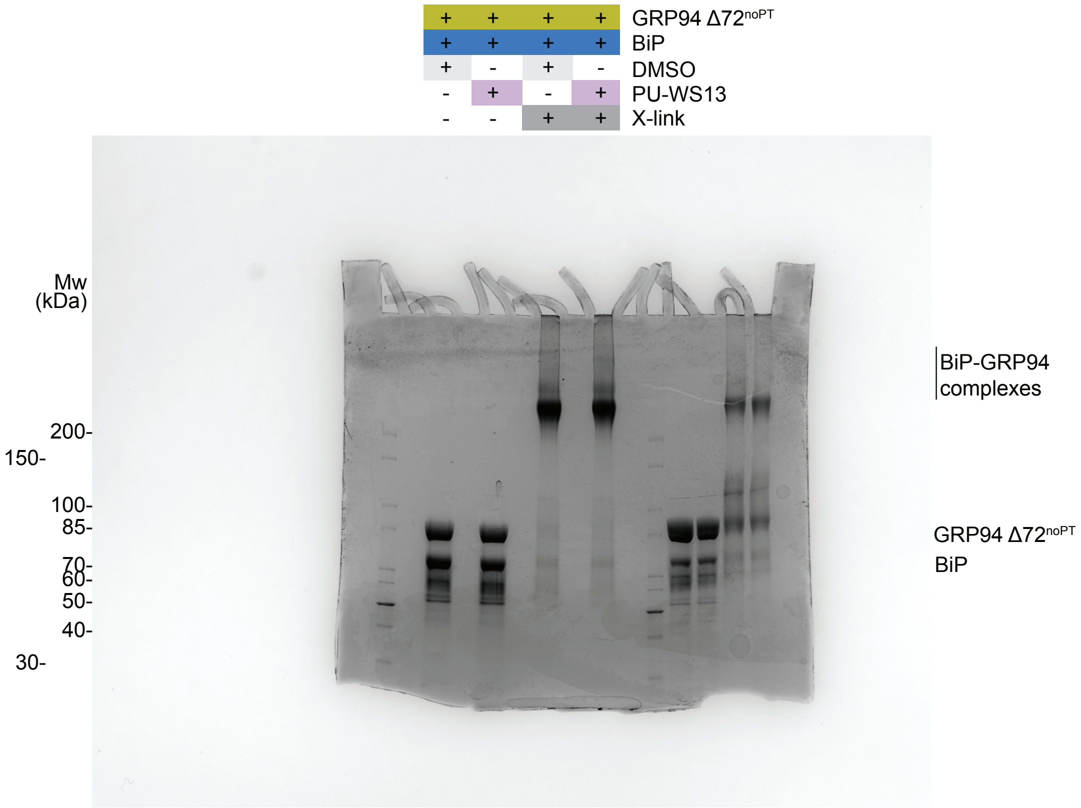

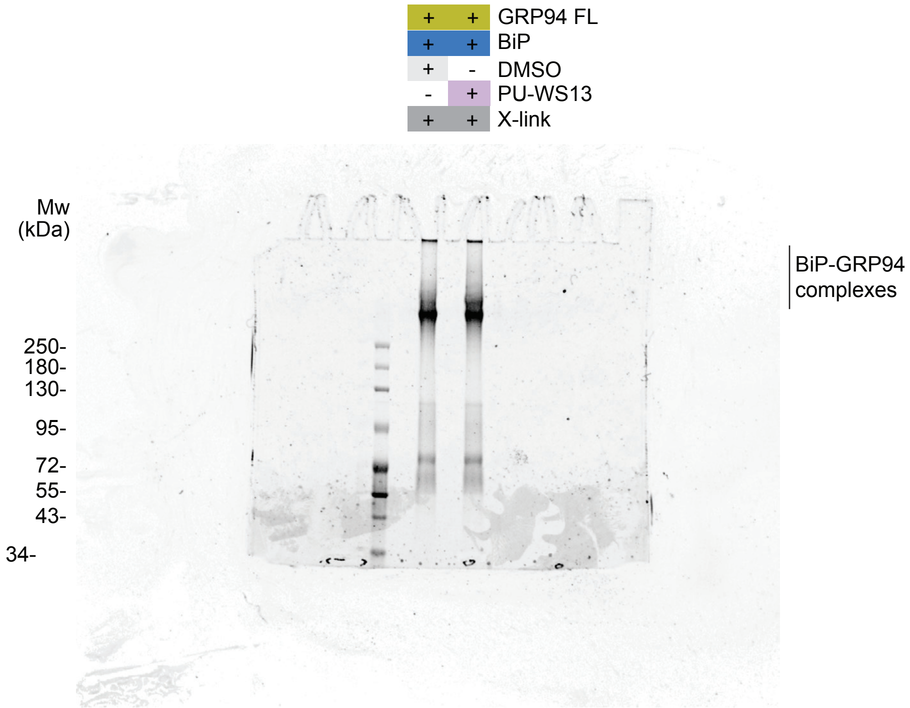

Data Source Extended Data Figure 5 cont.1

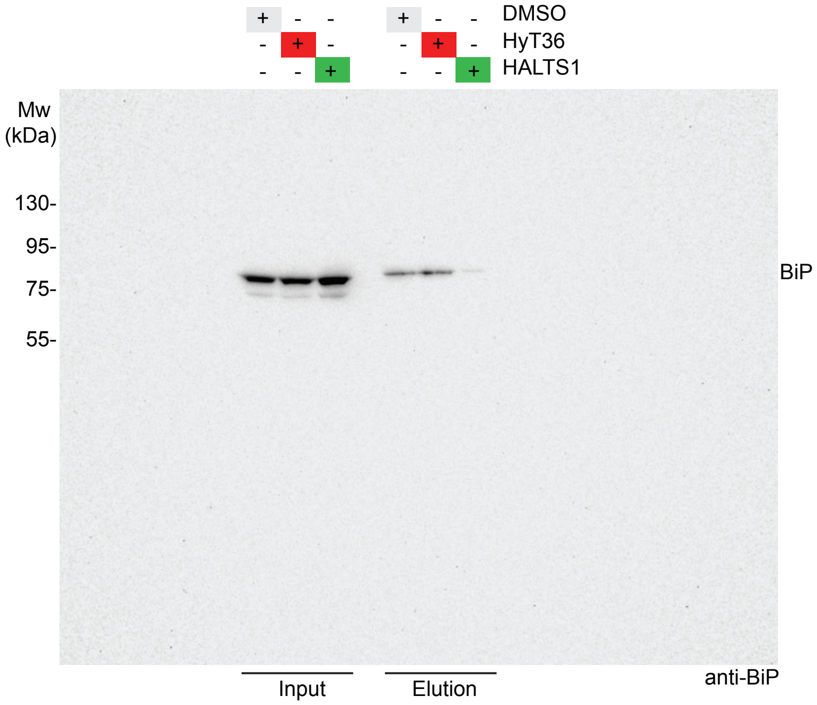

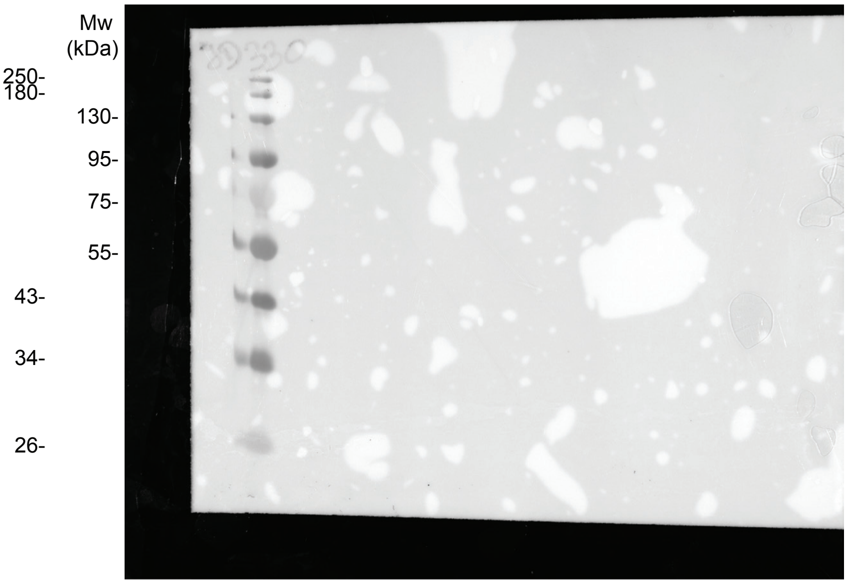

Data Source Extended Data Figure 5 cont.2

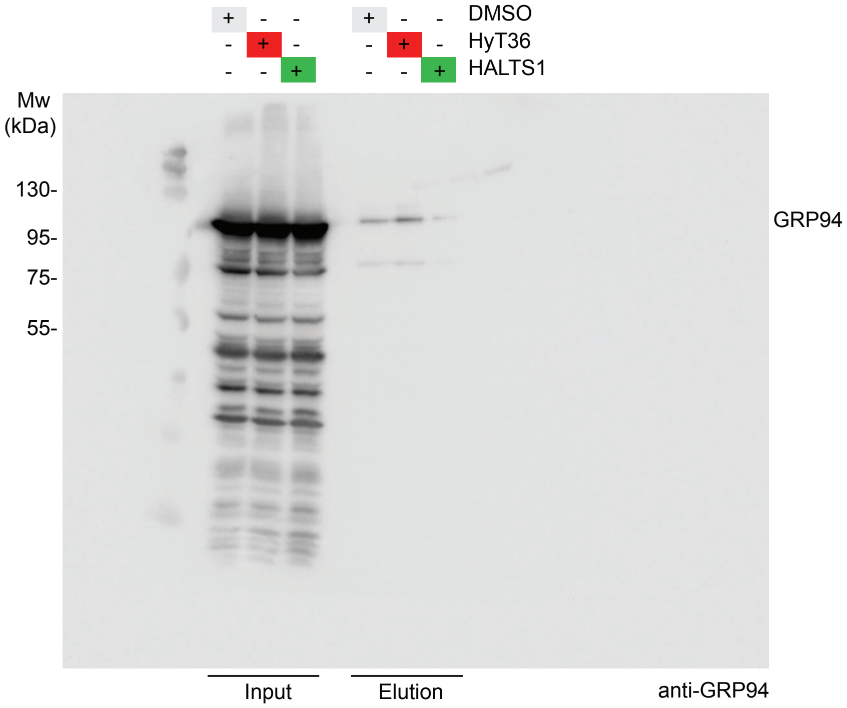

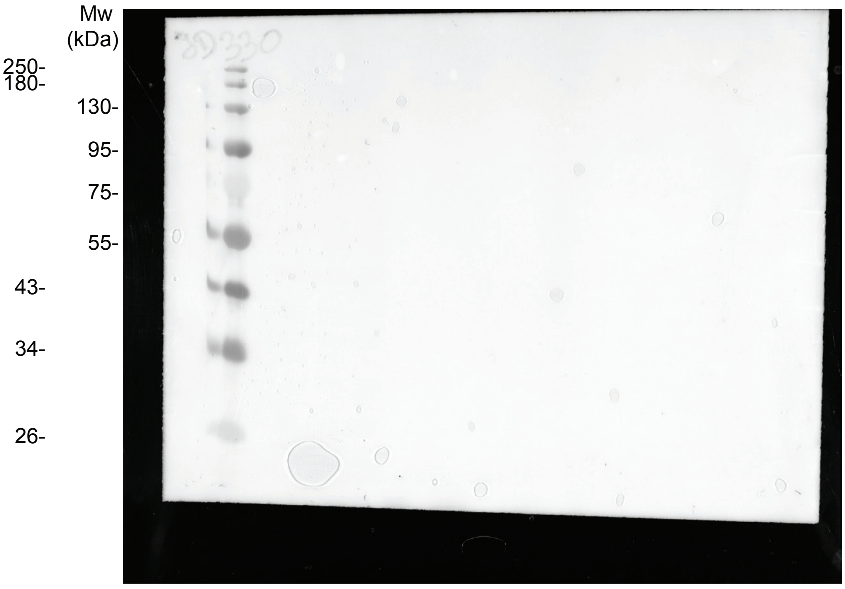

Data Source Extended Data Figure 5 cont.3

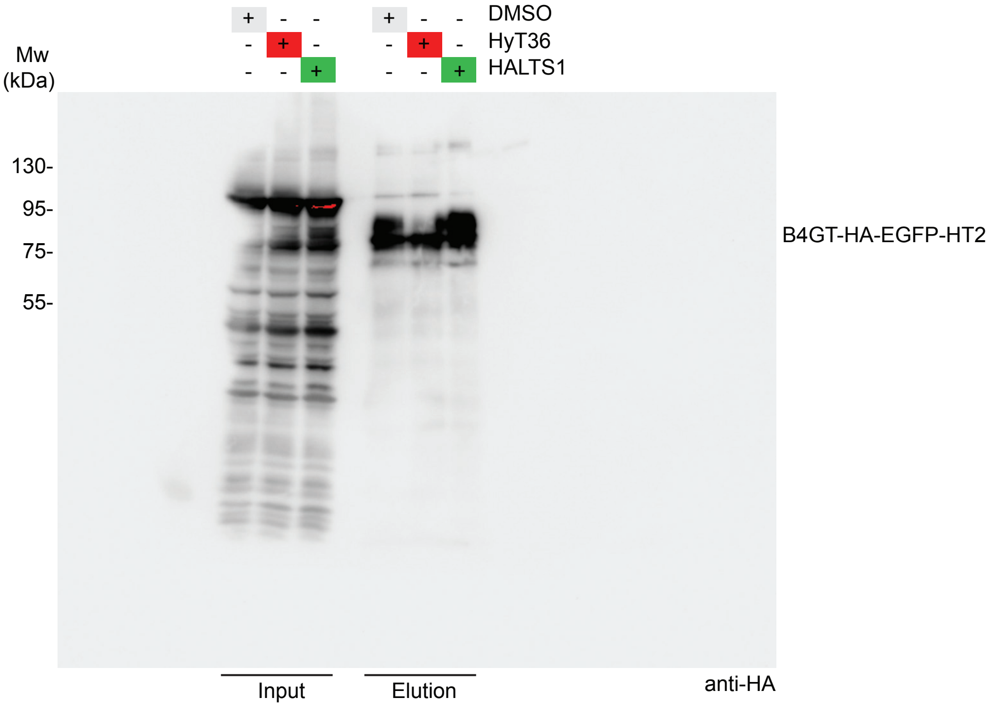

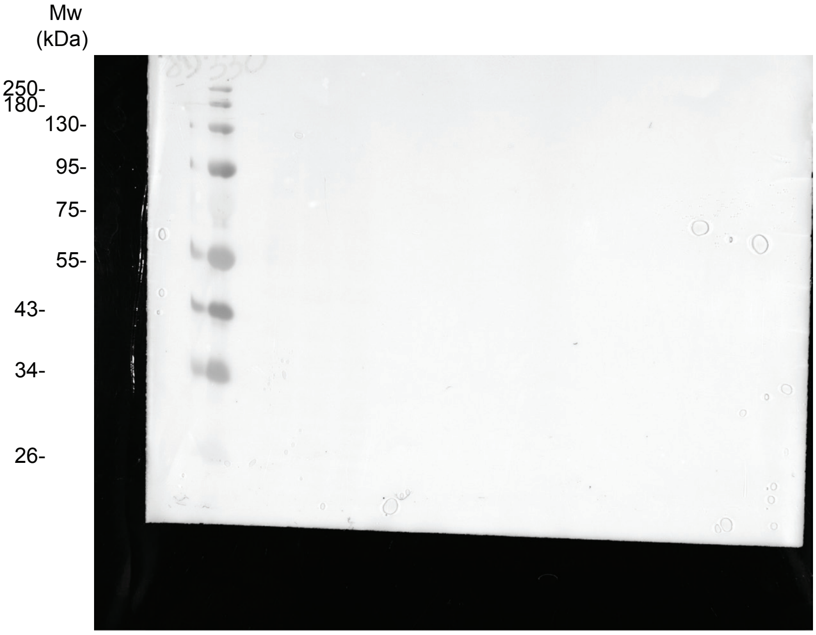

Data Source Extended Data Figure 6

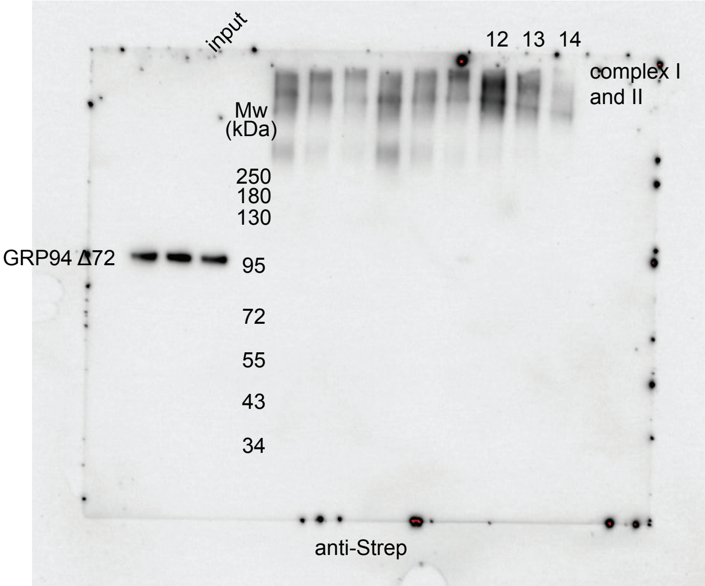

anti-Strep

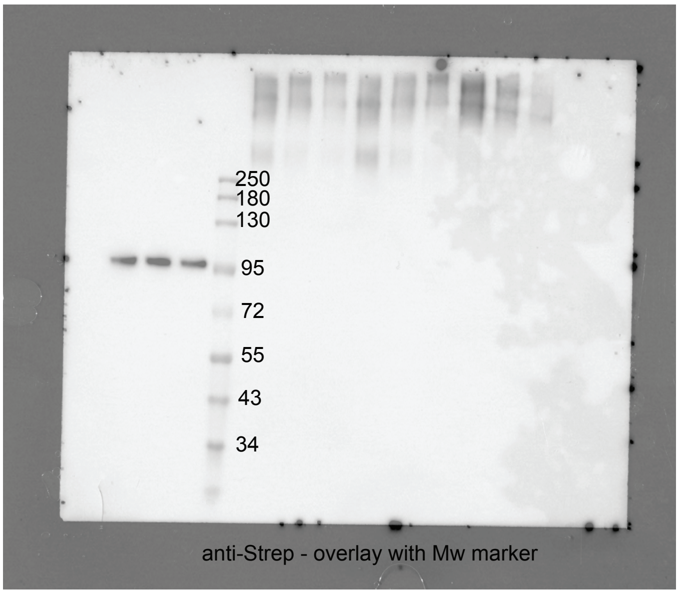

anti-Strep - overlay with Mw marker

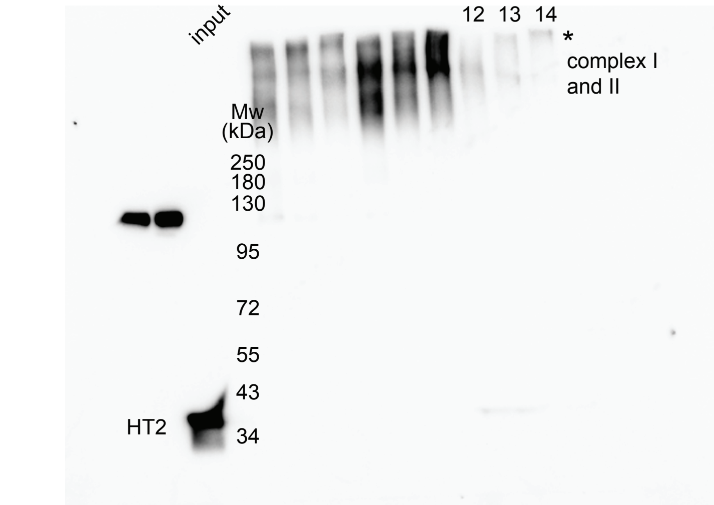

anti-HaloTag

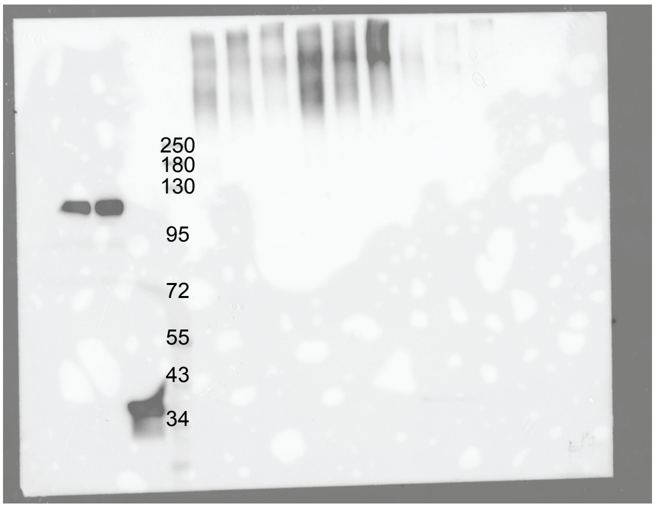

anti-HaloTag - overlay with Mw marker
